# Analyses of six homologous proteins of *Protochlamydia amoebophila *UWE25 encoded by large GC-rich genes (*lgr*): a model of evolution and concatenation of leucine-rich repeats

**DOI:** 10.1186/1471-2148-7-231

**Published:** 2007-11-16

**Authors:** Myriam Eugster, Claude-Alain H Roten, Gilbert Greub

**Affiliations:** 1 Center for Research on Intracellular Bacteria (CRIB), Institute of Microbiology, University Hospital Center and University of Lausanne, Switzerland; 2 Comparative Genometrics Center (CGC), Department of Fundamental Microbiology, University of Lausanne, Lausanne, Switzerland

## Abstract

**Background:**

Along the chromosome of the obligate intracellular bacteria *Protochlamydia amoebophila *UWE25, we recently described a genomic island Pam100G. It contains a *tra *unit likely involved in conjugative DNA transfer and *lgrE*, a 5.6-kb gene similar to five others of *P. amoebophila*:* lgrA *to *lgrD, lgrF*. We describe here the structure, regulation and evolution of these proteins termed LGRs since encoded by "Large G+C-Rich" genes.

**Results:**

No homologs to the whole protein sequence of LGRs were found in other organisms. Phylogenetic analyses suggest that serial duplications producing the six LGRs occurred relatively recently and nucleotide usage analyses show that *lgrB, lgrE *and *lgrF *were relocated on the chromosome. The C-terminal part of LGRs is homologous to Leucine-Rich Repeats domains (LRRs). Defined by a cumulative alignment score, the 5 to 18 concatenated octacosapeptidic (28-meric) LRRs of LGRs present all a predicted α-helix conformation. Their closest homologs are the 28-residue RI-like LRRs of mammalian NODs and the 24-meres of some *Ralstonia *and *Legionella *proteins. Interestingly, *lgrE*, which is present on Pam100G like the *tra *operon, exhibits Pfam domains related to DNA metabolism.

**Conclusion:**

Comparison of the LRRs, enable us to propose a parsimonious evolutionary scenario of these domains driven by adjacent concatenations of LRRs. Our model established on bacterial LRRs can be challenged in eucaryotic proteins carrying less conserved LRRs, such as NOD proteins and Toll-like receptors.

## Background

*Candidatus Protochlamydia amoebophila *UWE25 (hereafter named *P. amoebophila*) is an obligate intracellular symbiont infecting free-living amoebae [1]. Presenting significant gene sequence similarity with sequences of *Chlamydia *spp, this related bacterium exhibits a *Chlamydia-*like developmental cycle, which includes i) the proliferating reticulate body observed only within amoebae, ii) the infecting elementary body that may be found in amoebal vacuoles early after internalization, and iii) the crescent body considered as an additional infectious stage [2,3]. *P. amoebophila *belongs to the *Parachlamydiaceae *family, composed of, at least, two additional genera represented by *Parachlamydia acanthamoebae *[4] and *Neochlamydia hartmannellae *[5]*. Parachlamydia *was recently recognized as a novel agent of pneumonia (reviewed in [6,7]). Serological and molecular evidences suggested its role as an agent of community-acquired pneumonia [8-10] and aspiration pneumonia [11]. Moreover, we demonstrated that *Parachlamydia *may survive to human macrophages [12], by remaining somehow unrecognized from these major innate immune cells [13], by modulating the fate of the *Parachlamydia*-containing vacuole [14], and by inducing macrophage apoptosis [12].

The genome size of *P. amoebophila *is twice larger than the published genome sequences of *Chlamydiaceae *[15]. Preliminary genome analyses by Horn and coworkers showed the presence of transposases and of a cluster of high G+C content genes likely transferred horizontally [15]. The latter genes encode a type IV secretion system, supposed to be involved in the secretion of effector proteins [15,16]. However, Horn and coworkers did not identify *traA, traL, traK *and *traV*, and misannotated as *traF *an ORF similar to *trsF *carried by plasmid R391 of *Proteus rettgeri *[15,17].

After reannotation, identification of the four supplementary *tra *genes, and additional phylogenetic analyses based either on concatenated *tra *genes or on gene order comparison, we proposed that this type IV secretion system can play a role in conjugative DNA transfer and originated in proteobacteria [17]. The presence of similar *tra *operons on the pRF plasmid of *Rickettsia felis *[18] and on the circular chromosome of *Rickettsia bellii *[19], as well as the observation of sexual pili by electron microscopy of these two obligate intracellular bacteria [18,19] further supported that the gene products of the *tra *operon of *P. amoebophila *UWE25 are involved in conjugative DNA transfer. Furthermore, the concatenated *tra *genes of both rickettsia and *P. amoebophila *clustered in the maximum likelihood tree with a 95% bootstrap value [19].

In *silico *comparative genomics along this genome sequence enabled us to identify for the first time the genomic island Pam100G [17]. This inserted 100-kb mobile element is delimited by the presence of two *gly-tRNA *genes in tandem at its 5' end, and by direct repeats located at both ends [17]. Pam100G present a modular composition of its G+C content. The first module exhibits a G+C content (36.4%) similar to that of the host (36.1%) and contains a set of genes likely generated by chromosomal rearrangements. Then, three modules, which contain low G+C content (33.3–34.1%) phage-related genes, are intercalated with three high G+C content modules (38.7%–41.8%) [17]. The first G+C-rich module carried the type IV secretion system partially reported by Horn *et al*. [15]. The putative mobility of Pam100G as a conjugative plasmid was strongly supported by the identification of an episome carrying a similar *tra *unit during the genome sequence analyses of strain ATCC VR1471 of *Simkania negevensis*, a related bacterium (Myers G., oral presentation at the Chlamydia Basic Research Society Meeting, Indianapolis, 2005). The latter *tra *sequences are already available for BLAST analyses [20]. Interestingly, the genomic island of *P. amoebophila *also carries a huge 5.6-kb gene (pc1455). This gene, now called *lgrE*, is located 10 kb after the 3'-end of the *tra *unit (see below). It corresponds to the second high G+C content module (41.8%), suggesting it may, like the tra operon, have emerged in a common genomic environment similar to that of some alpha-, gamma-, delta- or epsilon-proteobacteria. This gene encodes one of the largest protein of *P. amoebophila *(1866 amino acids) exhibiting some similarities with the mammalian NOD3, a protein carrying LRR units [17].

The family of eucaryotic cytoplasmic proteins defined by a Nucleotide-binding Oligomerization Domain (NOD) [21] presents gene products exhibiting various functions ranging from regulators of apoptosis (such as Apaf-1) to proteins implicated in resistance against pathogens in mammals (NOD1, NOD2) and plants (R genes, R for resistance). Both R genes and mammalian NODs are composed of effector domains such as the CAspase Recruitment Domain (CARD) or the PYrin Domain (PYD, protein module defined by Bertin et al. [22] found in proteins that are thought to function in apoptotic and inflammatory signaling pathways), and of a carboxy-terminal leucine-rich repeat domain (LRRs) used in pathogen recognition. The NODs proteins also exhibit a NOD domain, which induces its self-oligomerization. LRR domains are concatenated repeats of 20- to 29-residue motifs present in all clades from viruses to eucaryotes. They have been classified in seven different subfamilies (reviewed in [23]). One of them, the ribonuclease inhibitor (RI)-like subfamily (RI-like LRR) is present in intracellular proteins of eucaryotes and exhibits the longest LRR motifs: 28–29 residues [23]. The LRRs of NOD proteins are the most studied RI-like LRRs. This ligand recognition domain is involved in the recognition of basic units of peptidoglycan, i.e. a common bacterial component: NOD1 recognizes the widely spread dipeptide γ-D-glutamyl-meso-diaminopimelate, and NOD2 the universal muramyl-L-alaninyl-D-glutamate, known as muramyl-dipeptide (MDP). The function of the NOD3 protein still remains unknown.

In this report, we described (i) the evolutionary history of the *lgrE *gene and of five paralogs (*lgrA*-*lgrD, lgrF*) present in the genome of *P. amoebophila*, (ii) the structure of the corresponding gene products and, finally, (iii) the structural and phylogenetic relationships existing between their LRR domains. Since almost no tools are available for molecular biology experiments on *Chlamydiales*, a putative regulation of these *lgr *genes and a possible role of these large proteins are proposed, based on various *in silico *analyses.

## Results and discussion

### *P. amoebophila *proteins homologous to LgrE

**Figure 1:**
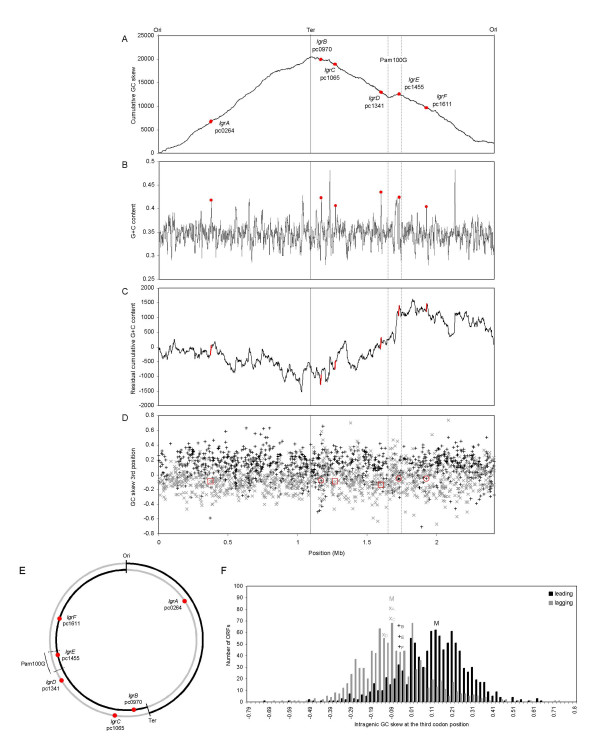
**A**: Position of the six *lgr *genes of *P. amoebophila *on the cumulative GC skew. **B**: on the G+C content curve (non-overlapping 1-kb windows). **C**: on the residual cumulative G+C content curve (5-kb windows sliding by 1-kb step). **D**: Values of the intragenic GC skew at the third position of the codons (GC_3_) versus the location of all 2031 ORFs of *P. amoebophila *encoded either by the leading (black +) or by the lagging strand (grey x), both strands defined by the origin and terminus of replication determined by the minimum and the maximum of the cumulative GC skew curve from Figure 1A; open red circles/squares highlight the *lgr*s located on the leading/lagging strand. **E**: Chromosome map of *P. amoebophila *showing by red circles the six *lgr*s encoded on the leading- (black), or the lagging strand (grey). **F**: Histogram of the GC_3 _values of all 2031 ORFs of *P. amoebophila *located on the leading (1065 ORFs) or lagging strand (988 ORFs). The values of the *lgr*s encoded by the leading/lagging strand are indicated by black +/grey x, and the median M of the leading/lagging strand values is labelled in black/grey. Since all six *lgr*s present higher G+C content than the rest of the genome (34.7%), they all exhibit steep slopes in the residual cumulative G+C content curve. The *lgrE *is located in Pam100G, a 100-kb genomic island presenting a particular GC skew profile whose boundaries are indicated by dashed lines in panels A to E. The GC_3 _values of all six *lgr*s is similar to that of the anti-orientated genes of *P. amoebophila*, although three *lgr*s are encoded by the leading strand (*lgrB, lgrE *and *lgrF*). Since GC_3 _values of the three latters (-0.08057 for *lgrB*, -0.05634 for *lgrE*, and -0.05228 for *lgrF*) are significantly lower than the median minus the standard deviation (0.129 ± 0.167) of all genes encoded on the leading strand (p = 0.005) and close to the median of the genes encoded by the lagging strand (median=-0.091 ± 0.171), it appears that an adaptation of the codon usage of these three *lgr*s is at work, due to a relatively recent re-orientation on the chromosome.

Using BLASTP, five additional large proteins homologous to the whole *lgrE *gene product were identified in the genome of *P. amoebophila *(Table 1). We named the six ORFs coded by large G+C rich genes *lgrA *to *lgrF *according to their position on the published chromosome sequence, starting from the putative origin of DNA replication indicated by GC skew analyses. Figures 1A and 1E show that these genes are scattered along the chromosome of the bacterium. As revealed by cumulative GC skew analyses (Figure 1A), *lgrE *is associated to the local inversion of the signal that highlights Pam100G, an already described genomic island [17].

**Table 1:** Characteristics of the six LGR proteins

Gene name	Protein number	Position	Strand	GC content (%)	Length [bp]	Length [aa]	Length of LRR region [aa]	Number of LRR	Identities with LgrE (BLASTP)
*lgrA*	pc0264	372491–377908	-	41.4	5418	1805	446	16	1590/1805 (88%)
*lgrB*	pc0970	1164574–1169391	-	42.6	4818	1605	239	8	1153/1600 (72%)
*lgrC*	pc1065	1267648–1272165	+	41.8	4518	1505	150	5	1104/1505 (73%)
*lgrD*	pc1341	1596253–1601547	+	43.1	5295	1764	411	15	1257/1776 (71%)
*lgrE*	pc1455	1726283–1731883	-	41.8	5601	1866	505	18	1866/1866 (100%)
*lgrF*	pc1611	1924358–1929142	-	40.9	4785	1594	225	8	1194/1597 (75%)

aa = amino acids

bp = base pairs

With more than 4500 nucleotides, the six *lgr *genes are among the 15 largest ORFs of the *P. amoebophila *genome. The presence of these six very similar ORFs, which likely originated by serial duplications from an unique ancestral gene, and not found in other sequenced bacteria, suggests that these proteins probably play an important role in the particular biology of these bacteria.

The genomic G+C content analysis displayed on Figure 1B shows that, with a G+C content ranging from 41.4 to 43.1%, the six *lgr*s present a G+C content higher than that of the average of the rest of the genome, suggesting a foreign origin. Due to a similar G+C content, all *lgrs* proteins might have a common origin with the *tra *operon, which most probably originated in proteobacteria [17]. Among the 2031 ORFs of *P. amoebophila*, 156 present a G+C content of more than 40%, including among others 28 ribosomal protein genes and all *lgr*s, the latters being the only high G+C ORFs encoding proteins larger than one thousand amino acids. As expected, all *lgr*s display a positive steep slope in the residual cumulative G+C content curve, due to their G+C content higher than the chromosome counterpart (Figure 1C). Of note, no particular gene environment of the *lgr*s could be highlighted by this analysis, except the already described genomic island, Pam100G, associated to *lgrE *[17].

All *lgr*s exhibit at the third codon position an enrichment in Cs characteristic of genes anti-oriented to chromosome replication [24], revealing that their common ancestor was most probably unique and anti-oriented. However, *lgrB, lgrE *and *lgrF*, are currently co-oriented (Figure 1D) and seem to exhibit an adaptation to this new relative position to chromosome replication, since their third codon position is slightly enriched in Gs (Figure 1E). Consequently, we may hypothesize that the currently co-oriented *lgrE *probably duplicated recently from the anti-oriented *lgrA *gene, and that *lgrB, lgrE *and *lgrF*, changed their orientation about at the same time, suggesting intense gene re-arrangements in a recent period during *P. amoebophila *speciation. The source of such genes rearrangements could be similar to the intense genetic exchanges revealed in Pam100G by the presence of the *tra *unit and the phage-related genes, i.e conjugative DNA transfer and/or transduction.

**Figure 2:**
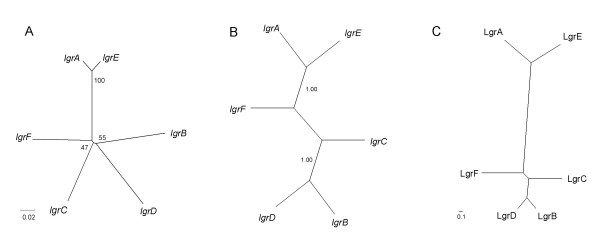
Phylogeny of the six LGR proteins encoding genes (*lgr*) of *P. amoebophila*. **A**: Neighbor-Joining tree of the nucleotide sequences of the *lgr*s without the sequence corresponding to the LRR domain, using the Kimura 2-parameter distance and the complete deletion parameter with a bootstrap analysis of 1'000 samples. Bootstrap values are shown in percent. **B**: Bayesian tree performed on the same non-LRR sequences was drawn after 100'000 generations, sampling 100 generations and 5 chains, respectively. The general-time reversible model was used with gamma rates of among-site variation. **C**: UPGMA tree based on the comparison of the amino acids composition of the LRRs: same data as those used for Figure 6 to calculate the center of gravity. In all three analyses, *lgrA *and *lgrE *are very close. Except in A, in which all other *lgr*s seem to emerge from the same nod, the relationships between the four others is strikingly conserved in the dendrograms B and C.

Furthermore, the six *lgr *genes show a very similar codon usage, not significantly different from the codon usage of most proteins of *P. amoebophila*. No particular relationships could be found by this particular analysis between *lgr*s and different kinds of genes: high G+C ORFs such as ribosomal protein genes, large proteins encoding genes or others (see Additional File 1(A)), and genes of the *tra *unit or the other genes of the genomic island (see Additional File 1(B)). Additional File 1(C) and its magnification (Additional File 1(D)) show that the variation of the codon usage is mainly due to the LRR domain (see below for precise delimitation). It also reveals that *lgrA *and *lgrE *present a very close codon usage suggesting that both genes result from a duplication more recent than the other *lgr *duplications. This hypothesis is confirmed by phylogenetic analyses performed on amino acids and nucleotides sequences with various phylogenetic methods (Neighbor-Joining [Figure 2A], Minimum Evolution and Maximum Parsimony [data not shown], Maximum Likelihood validated by Bayesian analyses [Figure 2B]).

### Overall structure of LGRs

The alignment of the six LGRs showed that their 1350 first amino acids are very similar (see Additional File 2). The more variable C-terminal part of these proteins presents gaps in the amino acid alignment, most of the latter measuring multiples of 28 residues, thus revealing an octacosapeptidic structure of this protein domain. This less conserved part of these proteins is characterized by the presence of concatenated Leucine-Rich Repeats (LRRs). The variation in the LRR number estimated after a precise delimitation of each unit (see below) almost fully explain the variable length of the six LGRs: the LRR domain varied from 168 to 515 amino acids while the remaining part of the proteins, designated by us as the non-LRR domain, present a very conserved length ranging from 1353 to 1369 amino acids (1360.5 ± 6.2).

Analyses of the secondary structure of these proteins with NNPREDICT (see Additional File 3(A)) showed a succession of α-helices (45.2–46.9%) and β-sheets (5.2–7.2%) in the LRR domain (see Additional File 4) precisely delimited by the cumulative alignment score (see below). In the non-LRR region (ca. 1350 first amino-acids), the proportion of α-helices and β-sheets are quite similar (42.0–46.6% and 5.8–7.7%, respectively). With the exception of LgrA and LgrE, the percentage of amino acids involved in α-helices in the carboxy-terminal region of LGR proteins defined as the LRR domain, is higher than in the rest of the protein (see Additional File 4). The recent duplication of LgrA and LgrE suggested by similarity in codon usage and by phylogenetic analyses (see above) is further supported by the very close secondary structure of these two proteins (Additional File 3(B)). Analyses performed by TMHMM v. 2.0 on the six LGR proteins identified no transmembrane domain. However, when looking for conserved Pfam motifs (see below the paragraph on the putative role of LGRs), we found a conserved domain present in mycoplasmal lipoproteins in LgrA, LgrB, LgrE and LgrF, suggesting that these LGRs exhibit a prokaryotic membrane attachment site probably acting as a membrane anchor [25].

Finally, we also analyzed the distribution of leucine within all six LGRs (see Additional File 5). While the leucine content of LGRs is higher (13.3 to 14.1%) than that of the other proteins encoded by *P. amoebophila *chromosome (11.7%), the leucine content of LGRs is not significantly different within the LRR- and non-LRR domains (13.2 to 17.3% and 13.1 to 13.5%). As expected, repetitions of the leucine pattern occur in the LRR domain at a periodic 28-residue interval.

### Other proteins of *P. amoebophila *exhibiting LRR domains: 23-, 25-, and 28-meres

LRR domains are defined as concatenated leucine-rich repeats units of 20–29 residues that generally fold into an arc or horseshoe shape [23]. BLASTP searches in the genome of *P. amoebophila *of proteins homologous to the LGRs highlights the presence of other LRRs proteins: (i) pc1145, a putative small protein exhibiting two repeats of 28 amino acids similar to the LRRs of the LGRs proteins, (ii) pc0038 containing 23-meric LRRs, and (iii) four additional proteins (pc1032, pc1462, pc1616 and pc0992) presenting 25-residue repeats. Further analyses based on the four latters detected 66 other proteins exhibiting relatively significant similarity to the four 25-meres. Our results describing 78 LRR-carrying proteins encoded on the chromosome of *P. amoebophila *UWE25 and the length of their LRRs extend the recently published preliminary analyses performed independently that identified 56 LRRs proteins in the genome of this strain [16].

### Common frame of the 28-meric LRRs determined by the Cumulative Alignment Score

Similarity analyses performed by BLAST allow rapid determination of LRR polypeptides in LGRs. Unfortunately, a common 28-meric frame shared by the six LGRs could not be proposed by this technique. Although the LRRs are abundantly discussed in the literature, there is no consensus on where each repeat precisely begins and ends. Therefore, we developed the Cumulative Alignment Score (CAS), a non a priori approach designed to unambigously define (i) the start of the LRR domain and (ii) the precise limits of the common unit frame of all LGRs and of other LRR-containing proteins of bacteria, animals and plants.

**Figure 3:**
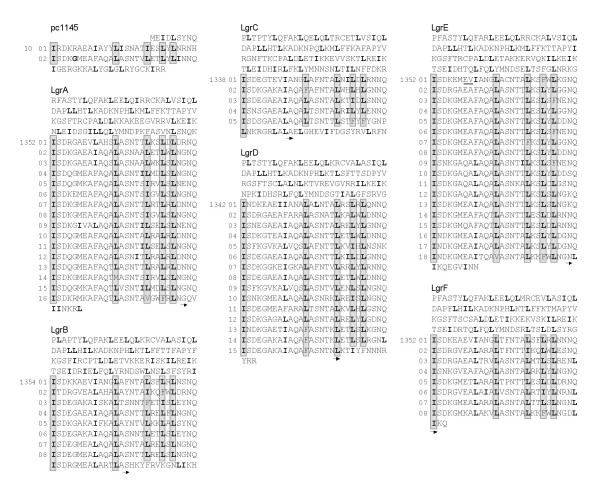
The 72 Leucine-Rich Repeats (LRRs) of pc1145 and of the six LGR proteins of *P. amoebophila*. The 28-residue motifs are aligned. The end of the protein is displayed for each LGR protein, starting from the four non-LRR 28-meres preceding the LRR domain. Leucine (L) and the related-isoleucine (I) are indicated in bold. While the common start of the LRR domain as defined by the CAS (Additional File 6) is indicated by the position of the first residue belonging to the LRR domain, the last residue is labeled by an arrow. Only LRR_1_^LgrE ^is larger (30 residues) than the 71 other 28-meric LRRs: also found in the first LRR of LgrA, LgrB, LgrC and LgrF, a dipeptide EV, underlined in LgrE, is duplicated in LRR_1_^LgrE^. All further analyses of the LGRs on the LRRs defined by the CAS are almost exclusively based on homologous 28-meric units and on repeats presenting similarities along at least two thirds in length of the 28-mere.

The representation of the protein sequences of the LGRs versus the CAS scores determined by preliminary common LRR sequences enabled us to precisely define the LRR domain of the six LGR proteins (see Additional File 6). Although the CAS of the six LGRs does not determine an entire number of repeats in the LRR domains, four of the six proteins present a LRR domain varying in length in LRR units by no more than three residues: LgrA, LgrD, LgrE and LgrF (Table 1, Figure 3). However, the final repeat LRR_15_^LgrD ^has a degenerated end and thus exhibits a lower similarity with the others. Therefore, the latter unit was not taken into account by the identity analysis of the CAS (Figure 3). All further analyses on the LRRs are based on repeats defined by the CAS by at least two thirds of the consensual residues.

The LRR domain of the LGR proteins are thus composed of 5 to 18 well conserved units of 28 amino acids composing 70 different LRRs (Figure 3, see Additional File 2). Furthermore, pc1145 also presents two 28-residue LRRs. Interestingly, only one out of these 72 LRRs differs in length: LRR_1_^LgrE^, the first LRR of LgrE, is a 30-residue unit, due to a tandem duplication of two codons (GAA-GTT-GAA-GTT) encoding the dipeptide Glu-Val present in the first LRR of all other LGRs, except LgrD. We confirmed the existence of this small duplication in LRR_1_^LgrE ^by resequencing this region, as previously described [26], but using the following primers: 5'CGGCTCCCTATATCAAAGGA and 5'GTTACCACCAAGGTAGAGTG.

**Figure 4:**
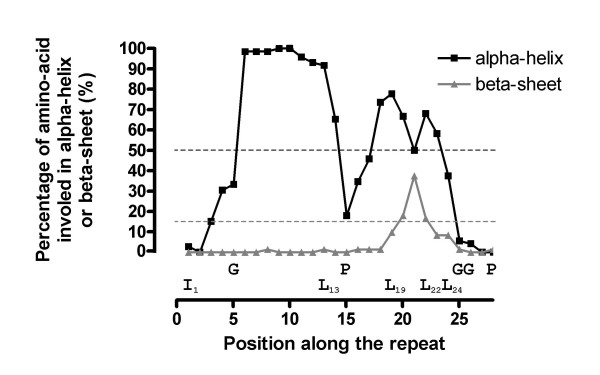
Common secondary structure of the LRRs of *P. amoebophila *defined by the CAS approach. On each position of the consensus of the 28-residue LRRs the proportion of amino acids of the 70 LRRs of the six LGR proteins predicted by NNPREDICT to belong to either an α-helix or a β-sheet is plotted. Perturbating amino acids present in some LRR, i.e. Glycyl (G) and Prolyl (P) residues are posted as they are located on the LRR unit. This figure clearly shows that while secondary structures are present within the LRR units, no particular structural configuration could be observed at the boundaries of the units.

The determination of the limits of the repeats of the LGRs performed by the CAS is unambiguously confirmed by the determination of a common frame for the six LGRs and by the presence of a conserved secondary structure within each repeat and no standard secondary structure at the LRR boundaries (Figure 4, see Additional Files 3 and 7). In several LRRs, amino acids present at the C-terminal part of the repeat were also predicted to be involved in a small β-sheet made of Leu_19_, Leu_22_, and Leu_24_. No other putative β-sheet was found elsewhere. Glycyl residues that could be part of a turn of the peptide backbone due to their non-stereospecific nature brought by the presence of the hydrogen atom as lateral chain, are always the 5^th ^amino acid of the repeats, except in LRR_1 _of all LGRs except LgrC, and in LRR_6 _and LRR_16 _of LgrA, and are often present at the 25^th ^or 26^th ^position (Figures 3 and 4). Interestingly, these glycyl residues are located at the boundaries of the consensual alpha-helix, thus not alterating the predominant secondary structure of the 28-residue unit, showing that structural constrains are imposed on the secondary structure of the LRRs. Moreover, only two prolyl residues are present in the 72 LRRs of the LGRs and of pc1145: one is the last residue of LRR_5_^LgrC ^and the other is the 15^th ^residue of LRR_5_^LgrF ^(Figure 4). The two prolines are located at positions of low α-helix signal. A definition of the protochlamydial 28-meric LRRs could thus be given: the secondary structure of these leucine-rich repeat motifs consists mainly of an α-helix located at the middle of each unit, and in some repeats, amino acids present at the distal part of the repeat were also predicted to be involved in a small β-sheet containing Leu_19_, Leu_22_, and Leu_24_.

### Evolutionary history of the LRR

**Figure 5:**
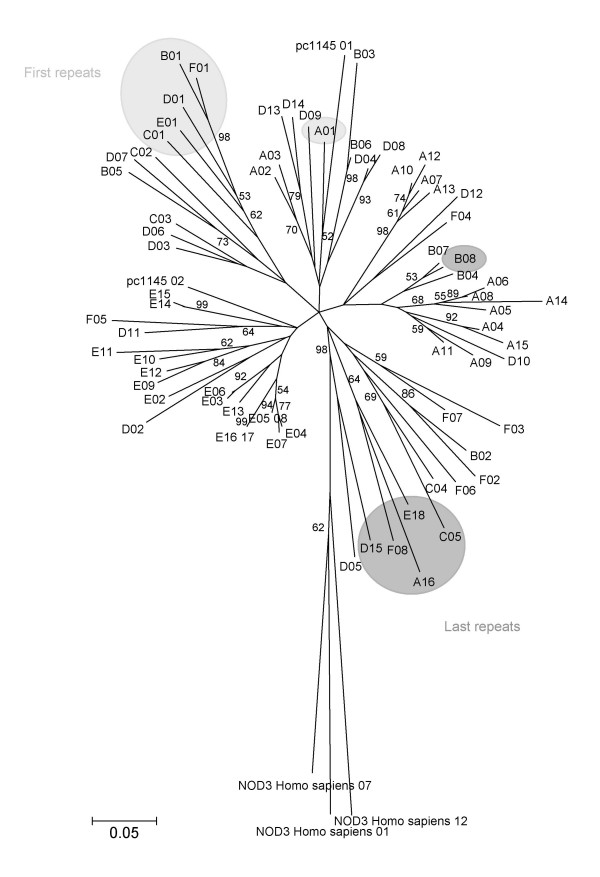
Neighbor-Joining tree inferred from the nucleotide sequence of the 72 LRRs related to LGRs of *P. amoebophila *and of three repeats of the human NOD3 protein. The p-distance model and the complete deletion parameter were performed with a 500-replicate bootstrap analysis. Only bootstrap values higher than 50% are shown.

**Figure 6:**
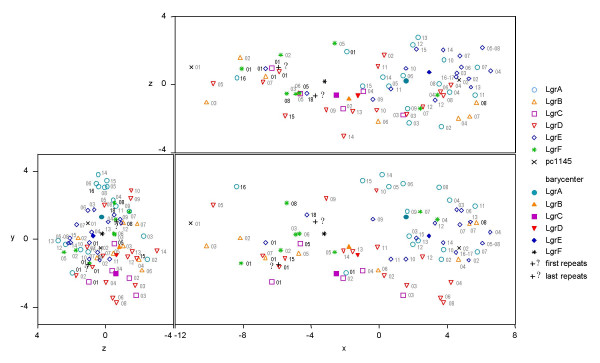
Three main dimensions of a principal coordinate analysis of the 72 LRRs related to LGRs of *P. amoebophila*. The distance is based on the identity of the amino acids of the repeats (Manhattan distance matrix). Filled symbols correspond to the center of mass of the repeats of the protein with the similar empty symbols. The barycenters of the six first repeats (α) and the six last repeats (ω) are indicated by + and the corresponding abbreviation. The first and the last repeats of the LGRs labelled in black tend to cluster together.

To determine how the LRRs were propagated within the LGR proteins, we inferred phylogenetic trees on nucleotide sequences of the 70 repeats of all LGRs and of the 2 repeats of pc1145 using the Neighbor-Joining method, the Kimura corrected p-distance (Figure 5) and a Bayesian method (Additional File 8(A)). We also drew phylogenic trees with an identity distance calculated from the proportion of conserved amino acids in pairs of repeats (see Additional File 8(B)). This comparison was also represented by a principal coordinate analysis (Figure 6).

These analyses showed that the first repeats of all LGRs except LgrA cluster together. On the Neighbor-joining (Figure 5), Bayesian (Additional File 8(A)) and UPGMA (Additional File 8(B)) phylogenetic trees, only three last LRRs of LGRs for which the CAS determined an almost entire last unit (LgrA, LgrE and LgrF) clustered together. This suggests that (i) the LRRs were probably multimerized after serial duplications of the LGR protein genes, (ii) that the repeats propagated independently in the six *lgr *genes by rearrangement/recombination of a few ancestral LRRs repeats within the LRR domains defined by the LRR units present at both ends, and (iii) all LGRs probably originated from an ancestral protein exhibiting a few ancestral LRR units able to produce a functional LRR domain.

The distance between all six barycenters (centers of gravity) on the two first dimensions of the PCO analysis was represented by an UPGMA tree (Figure 2C). Based on the LRRs domain, this tree was congruent with those inferred with the non-LRR part of LGR proteins (Figures 2A and 2B) and also confirmed that LgrA and LgrE duplicated recently.

**Figure 7:**
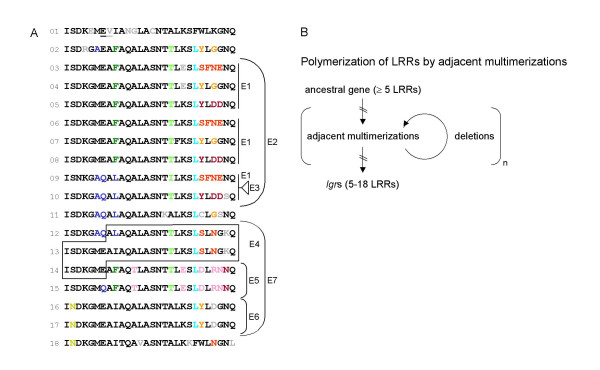
**A**: Sequence of the LRR domain of LgrE. The different motifs conserved in LRRs are highlighted in colour. Probable events of multimerization and deletion of repeats are indicated by E1 to E7 whose numbering is not chronological. A parcimonous scenario is proposed for the multimerization of the LRRs of LgrE. In the first part of the LRR domain: formation of a unit of three different LRRs (E1) that was later triplicated (E2) by two independent duplications in tandem. Finally, a deletion occurred (E3). On the second part of the LRR domain, a triplicate was build (E7), each member of it were duplicated (E4, E5 and E6). Our model shows that multimerization by adjacent concatenation occurred in LgrE. It also shows that LRR_1_^lgrE^, LRR_11_^lgrE^, and LRR_18_^lgrE ^were not involved directly during this process, suggesting more constrains on their sequences. Furthermore, it points out that the common frame is respected during all duplication events (E1, E2, E3, E5, E6, and E7) except one (E4). Finally, the later event (E4) associated to the deletion (E3) were certainly imposed by structural constrains acting on LRR_11_^lgrE^. **B**: General scheme of the mechanism of the multimerization of the LRRs of the LGRs.

Close inspection of the sequence of the LRRs of LgrE (Figure 3) showed that the variations between the repeats are locally distributed, suggesting that the multimerization of the LRRs occurred by serial adjacent duplications (Figure 7A). In order to determine if the repeats result from serial adjacent duplications, the identity scores between peptide sequences were calculated between each LRR of LgrE and i) its two closest repeats or ii) all other repeats of the protein. For all 18 repeats except LRR_2_^LgrE ^and LRR_8_^LgrE^, the average of the identity scores calculated on close neighbors is higher than the counterpart estimated between each of the 18 LRRs and all 17 others, showing that most LRRs are significantly more similar to their two closest LRRs than to all other repeats of the protein taken together (see panel A of Additional File 9(A)). This kind of analysis performed on the nucleotide sequences showed results very similar to those conducted on the amino acid sequences (panel B of Additional File 9(B)).

This identity analysis of the immediate LRR neighborhood was also performed by comparing the 28-meric units separated by 0 to 7 intercalary LRRs (Additional File 9(C)C). We observe on Additional File 9(C)C that immediate neighbors are the most similar. The observation performed with no intercalary sequence corresponds to those posted on additional Files 9AA and 9BB. This graph also shows that after immediate neighbors, the more similar are those separated by two units. In summary, the latter results suggest that LRR duplication occurred in the LRR domain of LgrE by inserting identical LRR units composed of one or three LRRs.

The average difference for the 18 LRRs of LgrE between the scores calculated on the immediate neighboring repeats and that estimated with all other repeats (1.6) was also calculated for the LRRs of the five other proteins, i.e. LgrA, LgrB, LgrC, LgrD and LgrF. It appeared that these values, although clearly lower, were all positive except for LgrF: 0.50, 0.14, 0.30, 0.10 and -0.21 respectively, thus confirming that for most LGRs the immediate neighbors of all LRRs are more similar than the others of the same LGR taken together.

### Evolution of LRRs by adjacent multimerization

As the LRRs of the LGRs are concatenates with no intervening sequences, we observed in LgrE that the LRR multimerization probably mainly results from adjacent duplications of DNA stretches of a multiple of 84 nucleotides encoding unique or multiple 28-residue LRRs sequences. These LRR multiplications produced by rearrangements/recombination are favored by the repetition of homologous sequences. Deletion could also appeared during rearrangement. Since a *recA *gene is present on the genome of *Protochlamydia amoebophila *UWE25 (pc1995), the RecA protein, which role in homologous recombination is abundantly documented [27,28], might be involved in these internal gene rearrangements. In first analysis, these recombination are independent of a particular 28-residue frame. This hypothesis was suggested by the initial alignments (see Additional File 2) showing that the LRRs of the six LGRs share no obvious common 28-residues frame. However, our non *a priori *approach CAS highlighting a common LRR frame clearly reveal that this common frame was generally respected by the recombination process during the *lrr *duplication.

These phylogenies and the detailed analysis of the sequence of the LRRs of LgrE enabled us to propose a parsimonious evolutionary scenario for a multimerization mechanism of these LRR domains. The LRRs of *P. amoebophila *seem to have originated from adjacent duplications and deletions of DNA stretches of a multiple of 84 nucleotides (Figure 7B).

### Evolutionary history of LGR neigbourhood of *P. amoebophila*

The six LGR proteins are very similar, particularly in the non-LRR domain and seem to result from relatively recent duplications in the history of these genes in the parachlamydial genome. To further investigate the evolutionary history of the six *lgr*s, BLAST searches of sequence homologous to the genes and intergenic sequences that flanked the *lgr*s were performed against the genome of *P. amoebophila*. Results are shown in Additional File 10. The narrow boxes of various sizes and colors represent the homologous regions present in the close environment of the six *lgr *genes. Interestingly, a homologous sequence of 633 bp is present on both sides of *lgrA *as an inverted repeat. This sequence belongs to a region that is also present downstream from *lgrC*, *lgrE *and *lgrF*. Another 360 bp inverted homologous sequence flanks *lgrB *on both side. Moreover, a shorter homologous region is present immediately upstream of the six *lgr *genes, that likely corresponds to promoter motifs.

### Prediction of promoter and terminator motifs

To find the promoter motifs of the six LGR proteins, we screened the 500 bp upstream of the translation start site for the presence of the so-called -35 and -10 conserved elements of the *Escherichia coli *canonical s70 promoter, which is also used by *Chlamydia *spp. [29,30]: the motifs TTG and TANNNT (the underlined nucleotides in the Table S2 are located at the position -35 and -12 of the translation start site), respectively, separated in *P. amoebophila *by 19 to 21 bp. We found 16 putative promoter motifs. Among them, five are very similar (all *lgr*s except *lgrC*) and are separated by 35–38 bp to the start codon of the five LGR encoding genes (see Additional File 11). A similar sequence is present before *lgrC *but its -35 motif differs from a single nucleotide (TTA instead of the consensual TTG). Calculated on the 50 last bp preceding the putative transcription start site, the G+C content of these six sequences ranges from 16.0 to 24.0%, confirming a putative regulatory nature. Moreover, the dinucleotide TG characteristic of promoter sequences [31] is starting at the -15 position. Other similar sequences of *lgrB, lgrC, lgrE *and *lgrF *are located at a distance of 152 to 157 bp of the start codon. Their G+C content on their last 50 bp range from 32.0 to 42.0%. The remaining seven sequences are not located at similar places before the different LGR encoding genes. The presence of two putative promoter motifs can reveal a complex regulation present in *P. amoebophila *to differentially regulate the expression of the *lgr *genes during different developmental stages. The presence of these promoter motifs strongly indicate that all *lgrs *are expressed genes. In addition, a putative terminator sequence was found in the 500 bp downstream of *lgrB*, using the FindTerm software.

### Proteins homologous to LGRs in other organisms

Using BLASTP and PSI-BLAST, proteins homologous to the LGR proteins of *P. amoebophila *were systematically searched in the non-redundant database (nr) maintained on the NCBI website [32]. A large number of proteins present in the genome of different organisms are significantly similar to the C-terminal part of the LGRs proteins corresponding to the last 150 to 505 amino-acids. This region of the LGRs exhibits 28-residue motifs very similar (178/418 = 41%) to the LRRs of the ribonuclease inhibitor (RI)-like subfamily, e.g. mammalian NOD3. The LRRs domain of LGRs also presents significant sequence similarity with DeliriumA (DlrA) of *Oryza sativa *and of *Dictyostelium discoideum *involved in the apoptosis [33,34] and with the LRRs of related genes of *Ralstonia solanacearum *and *Legionella pneumophila *(Additional File 12). BLASTN searches in the non-redundant database did not reveal any sequence homologous to LGRs.

Since a large number of proteins presented similarity in the LRR regions, we then performed additional BLASTN and BLASTP searches using the remaining parts of each LGR proteins. No proteins of other organisms is homologous to the non-LRR region of the LGRs excepted nine proteins of *D. discoideum *(E-value ranging from 2·10^-13 ^to 5·10^-2^). The latter matches found with the *D. discoideum *proteins corresponded to parts not characterized as catalytic sites or effector domains.

No additional hint was observed during other BLAST investigations (BLASTP and BLASTN) performed against all prokaryotic genomes as well as against those of *D. discoideum *and *Arabidopsis thaliana*. Since the chromosome sequence of *Simkania negevensis*, another intracellular amoebal chlamydia, is now available for similarity analyses, we were also interested to determine whether similar proteins were encoded in this genome by BLASTN, TBLASTN and TBLASTX analyses performed at the TIGR website [20]. No sequence larger than 300 nucleotides was identified showing significant similarities to LGRs. The smaller fragments that have been identified by BLASTN were similar to polypeptides located either in the LRR domain or in the remaining part of the LGRs.

### Relationship between LRRs domains of LGRs and of other organisms

We also applied the CAS method to delimit the LRR domains of other LRR proteins homologous to LGR identified by BLAST (Additional File 12). The same motif is conserved among all these LRR domains. The LRRs of mammals, plants and *D. discoideum *is composed of 28 amino acids, the LRR of *R. solanacearum *and *L. pneumophila *of 24 residues and those of *Tetrahymena thermophila *of 30-meric units. Determination of consensus LRR sequences for each protein showed that the repeats are well conserved in the LGRs of *P. amoebophila*, while they are more divergent within the LRR proteins of animals and plants (Additional File 12), indicating that the latters have a longer evolutionary history than the LGRs.

**Figure 8:**
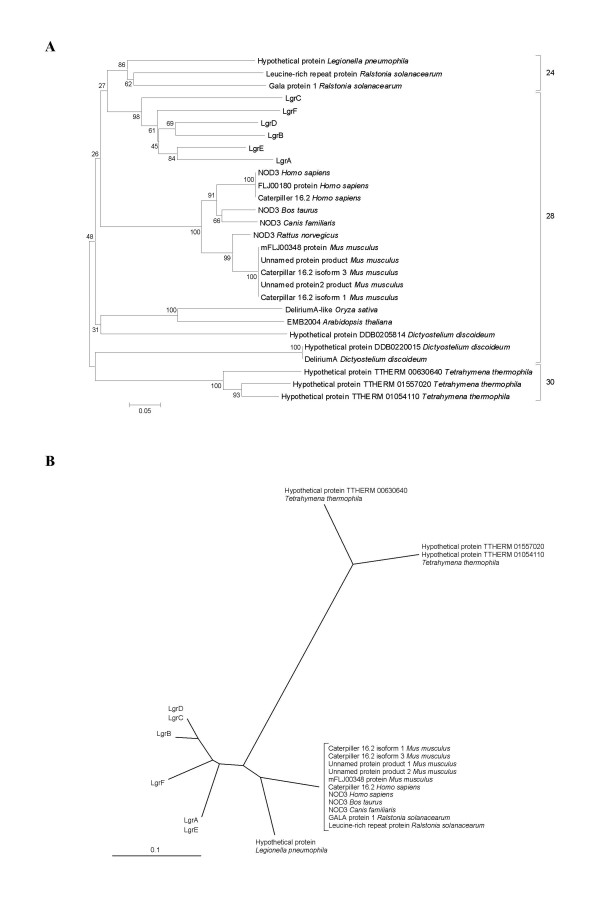
Phylogenetic analyses of the LRR domain of all the proteins presenting a similarity with an expect value < 10–50 with at least one of the six LGR proteins. **A**: Neighbor-Joining tree comparing the amino acids sequences by the corrected p-distance. Bootstraps of 1000 samples are shown in percent. **B**: UPGMA tree comparing the LRR consensus established on the same set of proteins. This analysis, which measures the identity of the amino acids, was performed only on the LRR consensus established on relatively well conserved repeats: 50% or more relative positions of the 28-mere presenting an identical amino acid in 50% or more of the LRRs of a given protein. Both analyses show that the closest relative of LRRs of LGRs are the bacterial 24-meric LRRs of *Legionella pneumophila *and related bacteria and the 28-meres of eucaryotes.

Phylogenetic analyses were performed on the LRR domains of the six LGR proteins and some of the LRR domain of the proteins found by BLAST. All proteins showing similarity (E-value < 10^-50^) with at least one of the six LGR proteins were included in the analyses (Figure 8A, see Additional File 13). Consequently, the LRRs domains of Toll and Toll-like proteins exhibiting not enough similarity with LGRs were not included in these analyses. Another phylogenetic tree was inferred on the LRR consensus established on each of these proteins presenting a high degree of similarity (Figure 8B). This analysis compared the consensual amino acids at each position and was performed only on the LRR domains that exhibit relatively well conserved repeats (at least 50% of consensual amino acids).

Both analyses indicated that the closest homologs of the 28-meric units of the LGRs seem to be carried by the 24-meres of the GALA protein 1 (a type III secretion system effector containing a LRR domain and a F-box domain, and considered essential for the virulence of the *R. solanacearum *in some plants [35]), of a LRR-protein of *Ralstonia solanacearum*, and of a hypothetical protein of *Legionella pneumophila*.

The next closest homologous LRRs to LRRs of the LGR were NOD proteins of mammals. The NODs of mammals exhibited the best matches in the BLAST searches. Moreover, analysis of the structure of the LRR domain of the LGR proteins showed that they probably belong to the same subfamily, LRR-RI. Like the motifs of LRRs of the LGR proteins, LRR-RI of mammals present 28-amino acid motifs.

### Putative roles of LGRs

We hypothesize that like LRR-RI of mammals, the protochlamydial LRR domain might be involved in bacterial recognition and that each LGR protein might present either slightly different effector domains or be able to recognize different bacterial motifs including nucleic acids. Since by BLAST analyses, we did not find any known effector domain in the non-LRR part of the six LGRs proteins, we searched for a putative effector domain by using the Pfam collection of multiple alignments of sequences determined by hidden Markov models (Additional File 14). All six LGRs exhibited three to four matches with the LRR_1 domain and one match with PetN (a small hydrophobic protein). LgrA, LgrB, LgrE and lgrF exhibited also a match with the lipoprotein_3 domain, present on a *Mycoplasma *protein and acting as an anchor, suggesting that these LGRs might be associated to membranes, despite the absence of transmembrane domain (see above). Interestingly, LgrA, LgrC, LgrD and lgrE present all a match with the DUF2027 domain, putatively involved in DNA mismatch repair. In addition, each LGRs proteins exhibited a few additional matches (see Additional File 14) that were mainly domains related with DNA metabolism. These putative active domains might thus be involved in the recombination necessary for the concatenation of the LRRs or might be essential in the recognition of foreign DNA.

It should be pointed out that *lgrE *is located near a *tra *operon likely involved in conjugative DNA transfer [17], which suggests that the LRR-RI motifs of LgrE might be involved in the recognition of a conjugative bacterial partner or in interactions with DNA/RNA molecules since eucaryotic LRR domains were shown to bind double helix of nucleotides [36].

Little is known on the biology of *P. amoebophila *UWE25. However, another related symbiont of amoebae, *Parachlamydia acanthamoebae*, was shown to resist destruction by macrophages, eliciting no oxidative burst and inducing nearly no secretion of proinflammatory cytokines [13]. Thus, LGR protein might alter the recognition of bacteria by the host cell by saturating recognition sites of the parachlamydial proteins secreted in the amoebal vacuoles containing these bacteria. However, the absence of genetic tools devoted to the study of the obligate intracellular *Chlamydiae *prevent further genetical investigations of the biological functions of these paralogous proteins. Since *Legionella *are facultative intracellular bacteria amenable to genetic manipulation and, like the *Parachlamydiaceae*, able to resist to both free-living amoebae and macrophages, it may be relevant to investigate the role of the *Legionella *LRR protein to understand the role of LGRs.

## Conclusion

In this work, we describe the evolutionary relationships existing between six large proteins encoded by homologous large G+C rich genes (*lgrA-lgrF*) of *P. amoebophila*. By analyzing the LRRs of these six homologous proteins of *Protochlamydia *amoebophila, we show that these repeats evolved by adjacent multimerization. Our model established on the bacterial 28-meric LRRs of the LGRs can now be challenged in related eucaryotic proteins composed of less conserved LRRs, such as NOD proteins and Toll-like receptors.

## Methods

### Genomic data

The complete genome sequence and the annotation file of *P. amoebophila *UWE25 (accession number: NC_005861) are available on the NCBI website [37]. The unfinished genome of strain ATCC VR1471 of *Simkania negevensis *was available for BLAST analyses at the TIGR website [20].

### BLAST analyses

Similarity analyses using BLASTP (BLOSUM62 matrix), iterative PSI-BLAST and BLASTN searches were performed by selecting default parameters against all available sequences in the non-redundant database available on the NCBI website [32,38]. Other BLASTP searches against all the prokaryote genomes, *D. discoideum *and *A. thaliana *were also conducted on the NCBI website [32] using the BLOSUM62 matrix. Moreover, BLASTN 2.2.13 without filter [38] and MegaBlast of coding and non-coding homologous sequence were performed along the genome of *P. amoebophila *UWE25 [37]. The unfinished genome of *S. negevensis *was also analyzed by BLASTN, TBLASTN and TBLASTX at the TIGR website [20] using default parameters. For all BLAST analyses, only matches presenting an expect value lower than 0.05 were considered significant.

### Pfam analysis

Unlike BLAST, hidden-Markov-models based proteins profile use a much intense probabilistic approach. Consequently, to search for conserved Pfam domains encoded by the *lgr*s genes, we performed the analyses using the Pfam 21.0 software against the database available on the Sanger website [39] using defaults parameter.

### Cumulative GC skew and intragenic GC skew at the third codon position

As initially inspired by Grigoriev *et al*. [40] and then applied by Roten *et al*. [41], a cumulative GC skew non-ponderated to the G+C content is a function Cm_GC_(i), measuring at each chromosome position *i *the excess of Gs by calculating the difference of the number of Gs and Cs, present from the first to the *i*-th nucleotide position:

Cm_GC_(i) = [Gi]- [Ci]

where Cm stands for cumulative. This cumulative GC skew analysis was performed on the complete genome sequence on non-overlapping 1-kb windows.

For each of the 2031 ORFs of *P. amoebophila*, the GC skew at the third codon position of a gene *j *Sk_GC3_(g_j_) was measured by calculating the difference between the frequency of Gs and Cs at the third codon position of the gene *j*, i.e. [Gg3_j_] and [Cg3_j_], normalized to the content of both nucleotides [24]:

Sk_GC3_(g_j_) = ([Gg3_j_] - [Cg3_j_])/([Gg3_j_] + [Cg3_j_])

### G+C content and residual cumulative G+C content analyses

The G+C content was calculated with 5-kb sliding windows moving in 1-kb steps. Derived from the GC profile approach [42], residual cumulative G+C content analysis reveals minor local variations of G+C content at the nucleotide level without being affected by windows of arbitrary size [17]. First, a cumulative G+C content curve is drawn by plotting at each chromosome position *i *the number of Cs and Gs from the first to the *i*-th position. Next, a linear regression is calculated, and finally a bidimensional graph is drawn on which chromosome positions on the horizontal axis are plotted versus the residues, i.e. the distances from each data to the linear regression, on the vertical axis. As already pointed out [17], while a flat segment on this curve reveals a DNA stretch locally exhibiting a G+C content similar to the chromosome counterpart, a segment presenting a positive or a negative slope indicates a region enriched or depleted in Cs and Gs, respectively.

### Alignment and phylogenetic analyses

The alignment of the six proteins encoded by Large G+C-Rich genes (*lgr*) was performed with ClustalW [43] in MEGA 3.1 [44] using default parameters. Neighbor-Joining, Minimum Evolution and Maximum Parsimony phylogenetic analyses were performed on amino-acid and nucleotide sequences of various datasets (six LGR proteins with or without related proteins identified by BLAST; either whole proteins or their LRR or non-LRR domains) with the same software using p-distance and the complete deletion option.

On the same datasets, we performed Bayesian analyses using MrBayes version 3.0b4, a program inferring Bayesian phylogenies [45,46]. The posterior probability validating the final tree is estimated using a Monte Carlo Markov Chain algorithm establishing a chain of possible dendrograms, which randomly wanders the tree space by sampling dendrograms until an equilibrium distribution is reached.

### Secondary structure prediction and transmembrane helices prediction

Prediction of the secondary structure of the LRRs were performed using two different softwares on the whole LGR sequences: i) NNPREDICT with the tertiary structure class option not selected [47,48], and ii) PREDATOR able to PREDict protein secondary structure- from a single sequence or a set of sequences [49,50].

Furthermore, the presence of putative transmembrane protein segments in the six LGRs were challenged by the TMHMM Server v. 2.0 designed for the prediction of TransMembrane helices based on a Hidden Markov Model [51,52]).

### Promoter and terminator detection

The 500 bp upstream of the six *lgr*s were screened for the presence of the *Escherichia coli *canonical σ^70 ^promoter. We searched the more conserved positions of the so-called -35 and -10 elements, i.e. the motifs TTG and TANNNT, separated by 19 to 21 bp [29,30], N representing a non-defined nucleotide.

The 500 bp downstream of the six *lgr*s were screened for the presence of terminator motifs with the software FindTerm designed for bacterial sequences [53].

### Determination of the common LRR frame by Cumulative Alignment Score (CAS)

For each LGR, we first defined by BLAST an initial 28-residue LRR consensus sequence by assigning at each of its 28 relative positions the amino acid present in at least half of all LRRs. Then, respecting the 28-residue frame used to determine the initial consensus, each amino acid of the LGRs is compared by an identity analysis to the consensus defined for each LGR: an alignment score is calculated by assigning 0 or 1 to each amino acid, respectively, different or identical to the amino acid of the consensus sequence. Finally, a Cumulative Alignment Score (CAS) curve is drawn by plotting to each amino acid position of the LGRs the sum of its alignment score to those of all preceding positions. On this representation, all six LGRs displayed a C-terminal steep slope region corresponding to the LRR region. Preliminary analyses showed that the limits of the LRR domains defined by our method are fully independent of the initial consensus frame. The CAS approach was able to unambiguously define a common frame to all LRRs of the six LGRs and thus to accurately define the LRR region of these proteins and of other proteins such as the mammalian NODs.

### Amino acid identity analyses on LRR sequences and related consensus

In addition to standard phylogenetic methods, we also compared the amino acid identity between pairs of any combination of the 72 LRRs of the six LGRs (70 units) and a small additional LRR protein (2 units). The divergence d_ab _between the LRRs a and b is calculated from the frequency of common amino acids c_ab _shared by both repeats at the same relative position of the polypeptide:

d_ab _= 1 - c_ab _

Such distances calculated for all pair combinations of the 72 LRRs are compared either by an UPGMA (Unweighted Pair Group Method with Arithmetic mean) dendrogram (Phylip 3.65; [54]) or bidimensionally using a principal coordinate analysis (see below).

This evolutionary distance based on identity was also used to compare within the LGRs the identity existing between a given LRR and two neighboring units concatenated to the LRR or separated by a same number of intercalary LRRs.

This approach was also used to compare the LRR consensus sequences specific to each protein. These comparisons were performed only on consensus sequences exhibiting a consensual amino acid in at least half of the 28 relative positions. The identity between a pair of given consensus LRR was defined as the number of consensual amino acids common to this pair divided by the number of consensual positions shared by both consensus sequences. The divergence between two LRR consensus sequences is calculated as above (equation 3). We then used these identity rates as evolutionary distances to infer a UPGMA tree.

### Multivariate comparisons: principal coordinate (PCO) and principal component analyses (PCA)

All PCO and PCA analyses were carried out with the software MVSP 3.1 [55]. Practically, both PCO and PCA represent the variability existing between n data in a n-dimension space. For each dimension, an eigenvalue is calculated. The bi/tridimensional graph able to best discriminate the data represents the n elements in the two/three dimensions exhibiting the highest eigenvalues [56]. PCA were chosen for codon usage analyses of all ORFs of *P. amoebophila *and PCO displaying a slightly more biased representation was selected for comparison of LRR since it was possible to replace Euclidean distances by Manhattan counterparts, the latter comparison exhibiting a slightly better data resolution.

## Authors' contributions

ME performed all the analyses of this work presented at University of Lausanne as her Master Thesis directed by GG (standard phylogeny, sequence alignment, protein secondary structure, biology of *Chlamydia*-like organisms) and co-supervised by CAHR (*in silico *comparative genomics, non-a priori approaches, LRR comparison tools, multivariate analyses, Bayesian phylogeny). ME drafted the manuscript that was improved and approved by all authors.

## Supplementary Material

Additional File 1 **Principal component analysis (PCA) of the codon usage of the 2031 ORFs of *P. amoebophila***. This analysis shows that *lgr*s present a codon usage similar to most other *P. amoebophila *ORFs. Click here for file

Additional File 2 **Alignment of the six LGR proteins of *P. amoebophila***. This alignment reveals how these proteins are closely related and detects a 28-residue period at the carboxy-terminal end of the sequences. Click here for file

Additional File 3 **Predicted secondary structure of the six LGRs**. This figure shows the secondary structure of the six LGR proteins of *P. amoebophila*, which is highly similar between LgrA and LgrE. Click here for file

Additional File 4 **Secondary structure of LGR proteins**. The data shown in this table are the percentage of the amino acids predicted to be involved in α-helixes and β-sheets. Click here for file

Additional File 5 **Leucine content of the six LGR proteins**. These analyses reveals no particular leucine enrichment of the LRR domain. Click here for file

Additional File 6 **Cumulative alignment score of the six LGR proteins**. Representation of the cumulative alignment score of LgrA to LgrF. Click here for file

Additional File 7 **Secondary structure of LRRs of the LGR proteins and related proteins**. This figure shows the secondary structure of the LRRs of LgrA to LgrF of *P. amoebophila*, of the human NOD3 protein and of a LRR-protein of *Legionella pneumophila*. Click here for file

Additional File 8 **Phylogeny of the 72 LRRs related to LGRs**. Phylogenetic analyses revealing that first and last repeats LRRs of LGRs proteins tend to cluster together. Click here for file

Additional File 9 **Identity scores between adjacent LRRs of the LGRs**. These figures reveal that adjacent LRRs are closely related. Click here for file

Additional File 10 **Immediate neighborhood of *lgrs***. This figure shows the local genetic map of the six *lgr *genes of *P. amoebophila*. Click here for file

Additional File 11 **Putative promoter motifs of the six *lgrs***. A list of putative promoter motifs present upstream of the six *lgr *genes of *P. amoebophila *is provided in this table. Click here for file

Additional File 12 **LRR consensus of the LGR proteins and of related proteins**. Table listing proteins presenting an identity (BLASTP) of less than 10^-50 ^with at least one of the LGR proteins and showing for each protein its LRR amino acid consensus. Click here for file

Additional File 13 **Phylogenetic analyses of the LRR domain of LGRs and related proteins**. Phylogenetic analyses showing the relatedness of LRRs of LGRs with LRR proteins of proteobacteria and NODs of mammals. Click here for file

Additional File 14 **Pfam analyses on all Lgrs**. Putative functions of the conserved Pfam domains of LGRs are shown in this table. Click here for file

## References

[B1] Collingro A, Toenshoff ER, Taylor MW, Fritsche TR, Wagner M, Horn M (2005). Candidatus *Protochlamydia amoebophila*, an endosymbiont of *Acanthamoeba *spp. Int J Syst Evol Microbiol.

[B2] Fritsche TR, Horn M, Wagner M, Herwig RP, Schleifer KH, Gautom RK (2000). Phylogenetic diversity among geographically dispersed *Chlamydiales *endosymbionts recovered from clinical and environmental isolates of *Acanthamoeba *spp. Appl Environ Microbiol.

[B3] Greub G, Raoult D (2002). Crescent bodies of *Parachlamydia acanthamoeba *and its life cycle within *Acanthamoeba polyphaga*: an electron micrograph study. Appl Environ Microbiol.

[B4] Amann R, Springer N, Schonhuber W, Ludwig W, Schmid EN, Muller KD, Michel R (1997). Obligate intracellular bacterial parasites of acanthamoebae related to *Chlamydia *spp. Appl Environ Microbiol.

[B5] Horn M, Wagner M, Muller KD, Schmid EN, Fritsche TR, Schleifer KH, Michel R (2000). *Neochlamydia hartmannellae *gen. nov., sp. nov. (*Parachlamydiaceae*), an endoparasite of the amoeba *Hartmannella vermiformis*. Microbiology.

[B6] Greub G, Raoult D (2002). *Parachlamydiaceae*: potential emerging pathogens. Emerg Infect Dis.

[B7] Corsaro D, Greub G (2006). Pathogenic potential of novel *Chlamydiae *and diagnostic approaches to infections due to these obligate intracellular bacteria. Clinical Microbiology Reviews.

[B8] Birtles RJ, Rowbotham TJ, Storey C, Marrie TJ, Raoult D (1997). *Chlamydia*-like obligate parasite of free-living amoebae. Lancet.

[B9] Marrie TJ, Raoult D, La Scola B, Birtles RJ, de Carolis E (2001). *Legionella*-like and other amoebal pathogens as agents of community-acquired pneumonia. Emerg Infect Dis.

[B10] Greub G, Berger P, Papazian L, Raoult D (2003). *Parachlamydiaceae *as rare agents of pneumonia. Emerg Infect Dis.

[B11] Greub G, Boyadjiev I, La Scola B, Raoult D, Martin C (2003). Serological hint suggesting that *Parachlamydiaceae* are agents of pneumonia in polytraumatized intensive care patients. Ann N Y Acad Sci.

[B12] Greub G, Mege JL, Raoult D (2003). *Parachlamydia acanthamoebae *enters and multiplies within human macrophages and induces their apoptosis. Infect Immun.

[B13] Greub G, Desnues B, Raoult D, Mege JL (2005). Lack of microbicidal response in human macrophages infected with *Parachlamydia acanthamoebae*. Microbes Infect.

[B14] Greub G, Mege JL, Gorvel JP, Raoult D, Meresse S (2005). Intracellular trafficking of *Parachlamydia acanthamoebae*. Cell Microbiol.

[B15] Horn M, Collingro A, Schmitz-Esser S, Beier CL, Purkhold U, Fartmann B, Brandt P, Nyakatura GJ, Droege M, Frishman D, Rattei T, Mewes HW, Wagner M (2004). Illuminating the evolutionary history of chlamydiae. Science.

[B16] Horn M, Collingro A, Schmitz-Esser S, Wagner M, Bavoil PM, Wyrick PB (2006). Environmental Chlamydia genomics. Chlamydia: genomics and pathogenesis.

[B17] Greub G, Collyn F, Guy L, Roten CA (2004). A genomic island present along the bacterial chromosome of the *Parachlamydiaceae *UWE25, an obligate amoebal endosymbiont, encodes a potentially functional F-like conjugative DNA transfer system. BMC Microbiol.

[B18] Ogata H, Renesto P, Audic S, Robert C, Blanc G, Fournier PE, Parinello H, Claverie JM, Raoult D (2005). The genome sequence of *Rickettsia felis *identifies the first putative conjugative plasmid in an obligate intracellular parasite. PLoS Biol.

[B19] Ogata H, La Scola  B, Audic S, Renesto P, Blanc G, Robert C, Fournier PE, Claverie JM, Raoult D (2006). Genome sequence of *Rickettsia bellii *illuminates the role of amoebae in gene exchanges between intracellular pathogens. PLoS Genet.

[B20] The Institute for Genomic Research (TIGR) website providing by BLAST access to TIGR's unfinished genomes sequences. http://tigr.org/.

[B21] Inohara N, Chamaillard M, McDonald C, Nunez G (2005). NOD-LRR proteins: role in host-microbial interactions and inflammatory disease. Annu Rev Biochem.

[B22] Bertin J, DiStefano PS (2000). The PYRIN domain: a novel motif found in apoptosis and inflammation proteins. Cell Death and Differentiation.

[B23] Kobe B, Kajava AV (2001). The leucine-rich repeat as a protein recognition motif. Curr Opin Struct Biol.

[B24] Lobry JR, Sueoka N (2002). Asymmetric directional mutation pressures in bacteria. Genome Biol.

[B25] The Pfam protein families database: description of *Mycoplasma *specific lipoproteins. http://pfam.jouy.inra.fr/cgi-bin/getdesc?name=Lipoprotein_3.

[B26] Casson N, Greub G (2006). Resistance of different *Chlamydia*-like organisms to quinolones and mutations in the quinolone resistance-determining region of the DNA gyrase A- and topoisomerase-encoding genes. Int J Antimicrob Agents.

[B27] Shibata T, DasGupta C, Cunningham RP, Radding CM (1980). Homologous pairing in genetic recombination: formation of D loops by combined action of RecA protein and a helix-destabilizing protein. Proc Natl Acad Sci USA.

[B28] Fulconis R, Mine J, Bancaud A, Dutreix M, Viovy JL (2006). Mechanism of RecA-mediated homologous recombination revisited by single molecule nanomanipulation. EMBO J.

[B29] Timms P, Towsey M, Hogan J, Mathews S, Max Chernesky et al (2006). Whole genome transcript start site and promoter prediction in *Chlamydia*. Chlamydial Infections: Proceedings of the Eleven International Symposium on Human Chlamydial Infections.

[B30] Hefty PS, Stephens RS (2007). Chlamydial type III secretion system is encoded on ten operons preceded by sigma 70-like promoter elements. J Bacteriol.

[B31] Helmann JD (1995). Compilation and analysis of *Bacillus subtilis *sigma A-dependent promoter sequences: evidence for extended contact between RNA polymerase and upstream promoter DNA. Nucleic Acids Research.

[B32] Website of the National Center for Biotechnology Information (NCBI). http://www.ncbi.nlm.nih.gov/.

[B33] Adam M, Levraud JP, Golstein P, Williams J, Weijer K, Schaap P (2000). Delirium A, not such a crazy mutant. Proceedings of the International Dictyostelium Conference.

[B34] Levraud JP, Adam M, Luciani MF, Dubus-Bonnet V, Roisin-Bouffay C, Golstein P, Firtel RA, Loomis WL (2001). Genetic and morphological analysis on *Dictyostelium *cell death. Proceedings of the International Dictyostelium Conference.

[B35] Angot A, Peeters N, Lechner E, Vailleau F, Baud C, Gentzbittel L, Sartorel E, Genschik P, Boucher C, Genin S (2006). *Ralstonia solanacearum *requires F-box-like domain-containing type III effectors to promote disease on several host plants. Proc Natl Acad Sci USA.

[B36] Bell JK, Botos I, Hall PR, Askins J, Shiloach J, Segal DM, Davies DR (2005). The molecular structure of the Toll-like receptor 3 ligand-binding domain. Proc Natl Acad Sci USA.

[B37] Genome of Candidatus *Protochlamydia amoeophila *strain UWE25.. http://www.ncbi.nlm.nih.gov/sites/entrez?Db=genome&Cmd=ShowDetailView&TermToSearch=399.

[B38] Altschul SF, Madden TL, Schäffer AA, Zhang J, Zhang Z, Miller W, Lipman DJ (1997). Gapped BLAST and PSI-BLAST: a new generation of protein database search programs. Nucleic Acids Res.

[B39] Sanger website for Pfam analyses. http://www.sanger.ac.uk/Software/Pfam/.

[B40] Grigoriev A (1998). Analyzing genomes with cumulative skew diagrams. Nucleic Acids Res.

[B41] Roten CA, Gamba P, Barblan JL, Karamata D (2002). Comparative Genometrics (CG): a database dedicated to biometric comparisons of whole genomes. Nucleic Acids Res.

[B42] Zhang R, Zhang CT (2004). A systematic method to identify genomic islands and its applications in analyzing the genomes of *Corynebacterium glutamicum *and *Vibrio vulnificus *CMCP6 chromosome I. Bioinformatics.

[B43] Thompson JD, Higgins DG, Gibson TJ (1994). CLUSTAL W improving the sensitivity of progressive multiple sequence alignment through sequence weighting, position-specific gap penalties and weight matrix choice. Nucleic Acids Res.

[B44] Kumar S, Tamura K, Nei M (2004). MEGA3: Integrated software for Molecular Evolutionary Genetics Analysis and sequence alignment. Briefings in Bioinformatics.

[B45] Ronquist F, Huelsenbeck JP (2003). MrBayes 3: Bayesian phylogenetic inference under mixed models. Bioinformatics.

[B46] Huelsenbeck JP, Ronquist F (2001). MrBayes: Bayesian inference of phylogenetic trees. Bioinformatics.

[B47] NNPREDICT website: secondary structure analyses of proteins. http://www.cmpharm.ucsf.edu/~nomi/nnpredict.html.

[B48] Kneller DG, Cohen FE, Langridge R (1990). Improvements in protein secondary structure prediction by an enhanced neural network. J Mol Biol.

[B49] PREDATOR website: secondary structure analyses of proteins. http://bioweb.pasteur.fr/seqanal/interfaces/predator-simple.html.

[B50] Frishman D, Argos P (1996). Incorporation of long-distance interactions into a secondary structure prediction algorithm. Protein Engineering.

[B51] TMHMM Server v. 2.0 designed for the prediction of TransMembrane helices. http://www.cbs.dtu.dk/services/TMHMM-2.0/.

[B52] Krogh A, Larsson B, von Heijne G, Sonnhammer EL (2001). Predicting transmembrane protein topology with a hidden Markov model: application to complete genomes. J Mol Biol.

[B53] FindTerm Software: detection of gene terminators. http://sun1.softberry.com/berry.phtml?topic=findterm&group=programs&subgroup=gfindb.

[B54] Felsenstein J (1989). PHYLIP-Phylogeny inference package. Cladistics.

[B55] Software MVSP 3.1: multivariate analyses. http://www.kovcomp.com/mvsp/index.html.

[B56] Gower JC (1966). Some distance properties of latent root and vector methods used in multivariate analysis. Biometrika.

[B57] Prefixes used in chemistry. http://home.comcast.net/~igpl/NWC.html.

