# Supplementary material for: Analyses of six homologous proteins of Protochlamydia amoebophila UWE25 encoded by large GC-rich genes (lgr): a model of evolution and concatenation of leucine-rich repeats
Source: BMC Evol Biol. 2007 Nov 16;7:231. doi: 10.1186/1471-2148-7-231 (PMC2216083; doi:10.1186/1471-2148-7-231)
Supplement: Additional File 1 — Principal component analysis (PCA) of the codon usage of the 2031 ORFs of P. amoebophila. This analysis shows that lgrs present a codon usage similar to most other P. amoebophila ORFs. [file 1471-2148-7-231-S1.pdf]

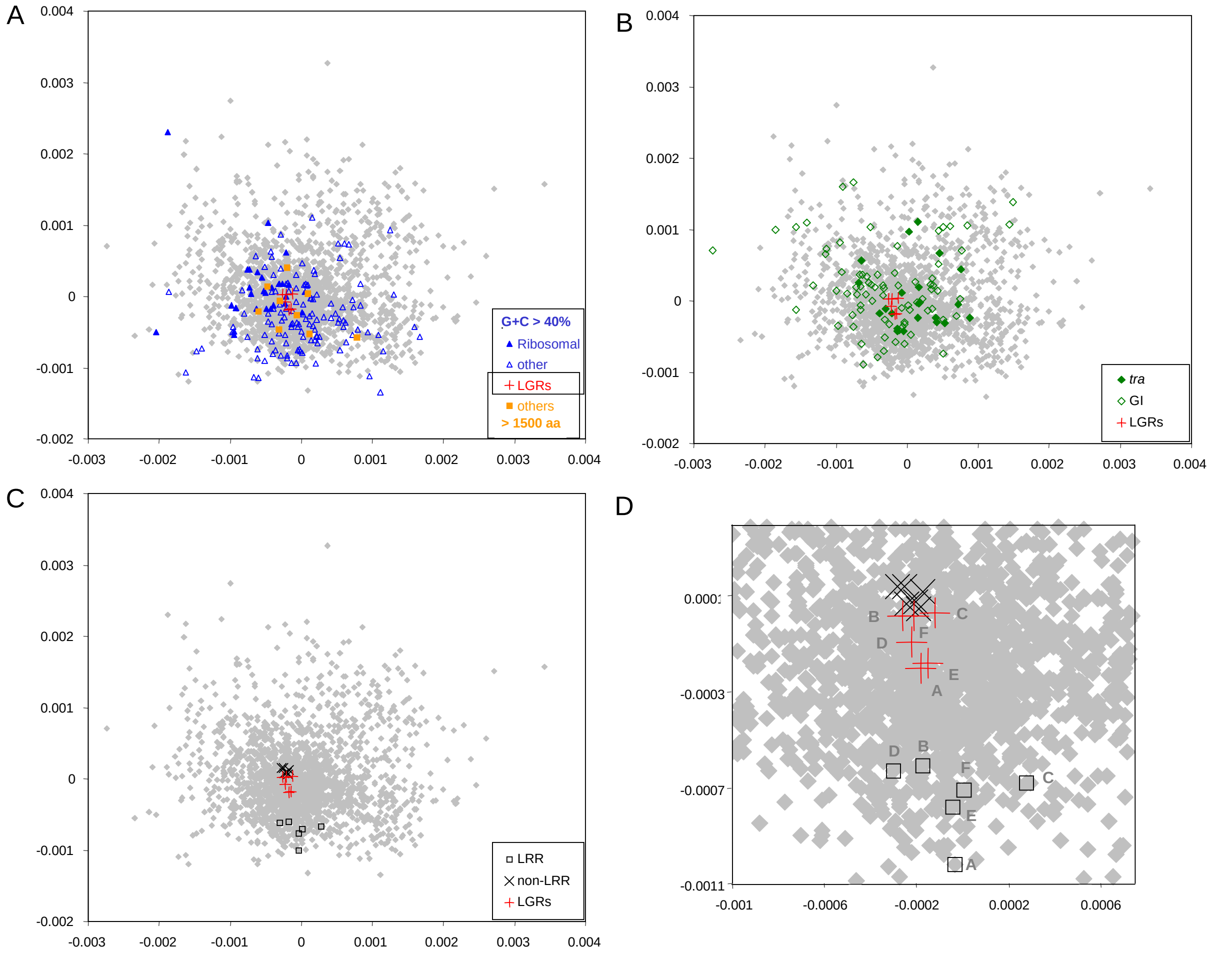

Additional file 1.
Principal component analysis (PCA) of the codon usage of the 2031 ORFs of *P. amoebophila* performed on Euclidean distances. The *lgr*s, the ORFs exhibiting a G+C content higher than 40%(G+C > 40%), the ribosomal genes (ribosomal), the genes encoding proteins larger than 1500 residues (> 1500 aa) (**A**); all genes belonging to the Pam100G genomic island (GI) and to the *tra* operon (*tra*) (**B**). The LRR domain (LRR) and the remaining part of the *lgr*s (non-LRR) (**C**) are independantly highlighted. Panel **D** is an higher magnification of panel C. The protein genes presenting a common codon usage in *P. amoebophila* would be found around the coordinates (0,0). In this particular analysis, no particular codon usage could be assigned to the *lgr*s.
