# Supplementary material for: Analyses of six homologous proteins of Protochlamydia amoebophila UWE25 encoded by large GC-rich genes (lgr): a model of evolution and concatenation of leucine-rich repeats
Source: BMC Evol Biol. 2007 Nov 16;7:231. doi: 10.1186/1471-2148-7-231 (PMC2216083; doi:10.1186/1471-2148-7-231)
Supplement: Additional File 2 — Alignment of the six LGR proteins of P. amoebophila. This alignment reveals how these proteins are closely related and detects a 28-residue period at the carboxy-terminal end of the sequences. [file 1471-2148-7-231-S2.ppt]

## Slide 1
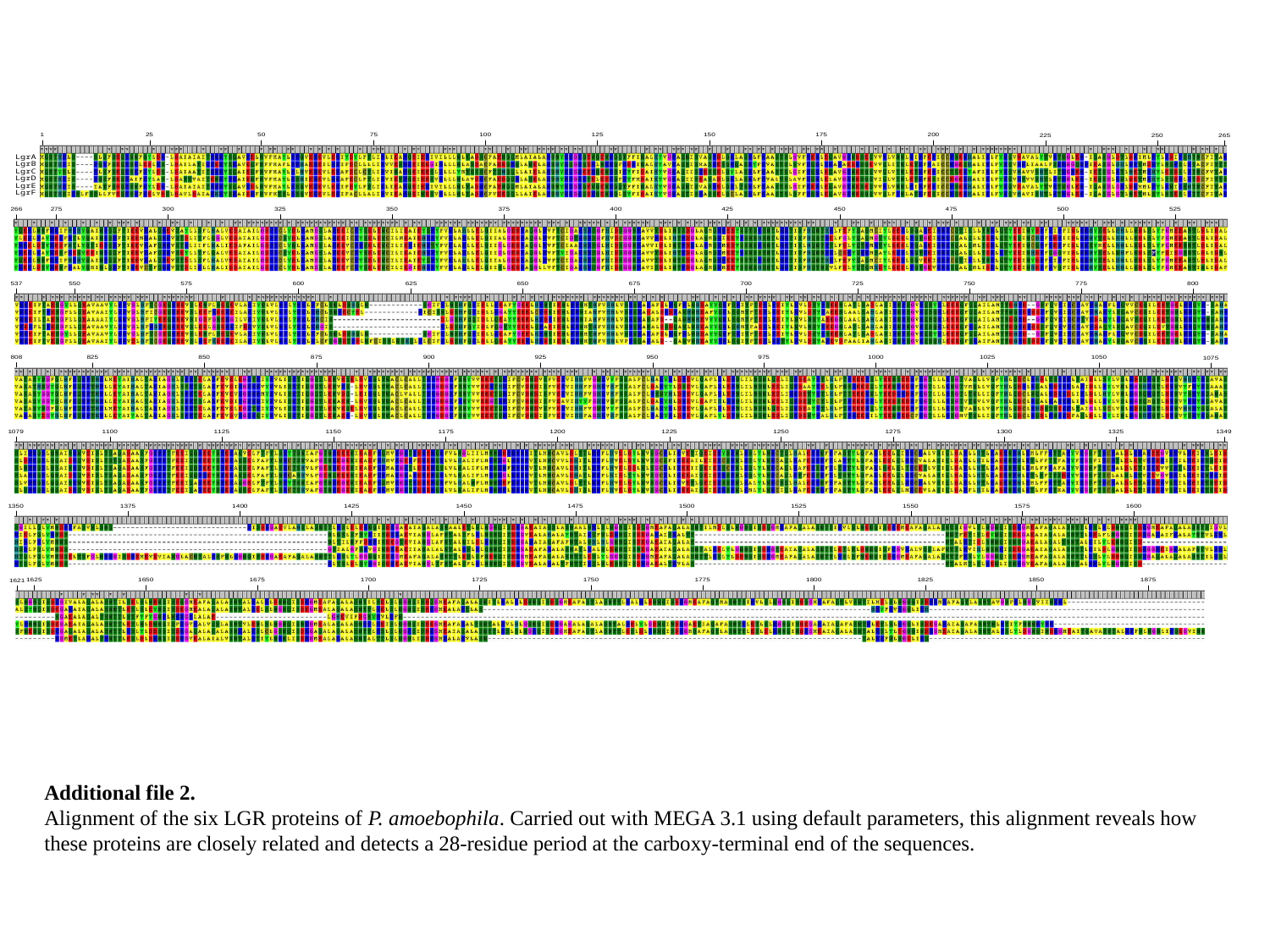

Additional file 2.
Alignment of the six LGR proteins of P. amoebophila. Carried out with MEGA 3.1 using default parameters, this alignment reveals how these proteins are closely related and detects a 28-residue period at the carboxy-terminal end of the sequences.
