# Supplementary material for: Analyses of six homologous proteins of Protochlamydia amoebophila UWE25 encoded by large GC-rich genes (lgr): a model of evolution and concatenation of leucine-rich repeats
Source: BMC Evol Biol. 2007 Nov 16;7:231. doi: 10.1186/1471-2148-7-231 (PMC2216083; doi:10.1186/1471-2148-7-231)
Supplement: Additional File 3 — Predicted secondary structure of the six LGRs. This figure shows the secondary structure of the six LGR proteins of P. amoebophila, which is highly similar between LgrA and LgrE. [file 1471-2148-7-231-S3.ppt]

## Slide 1
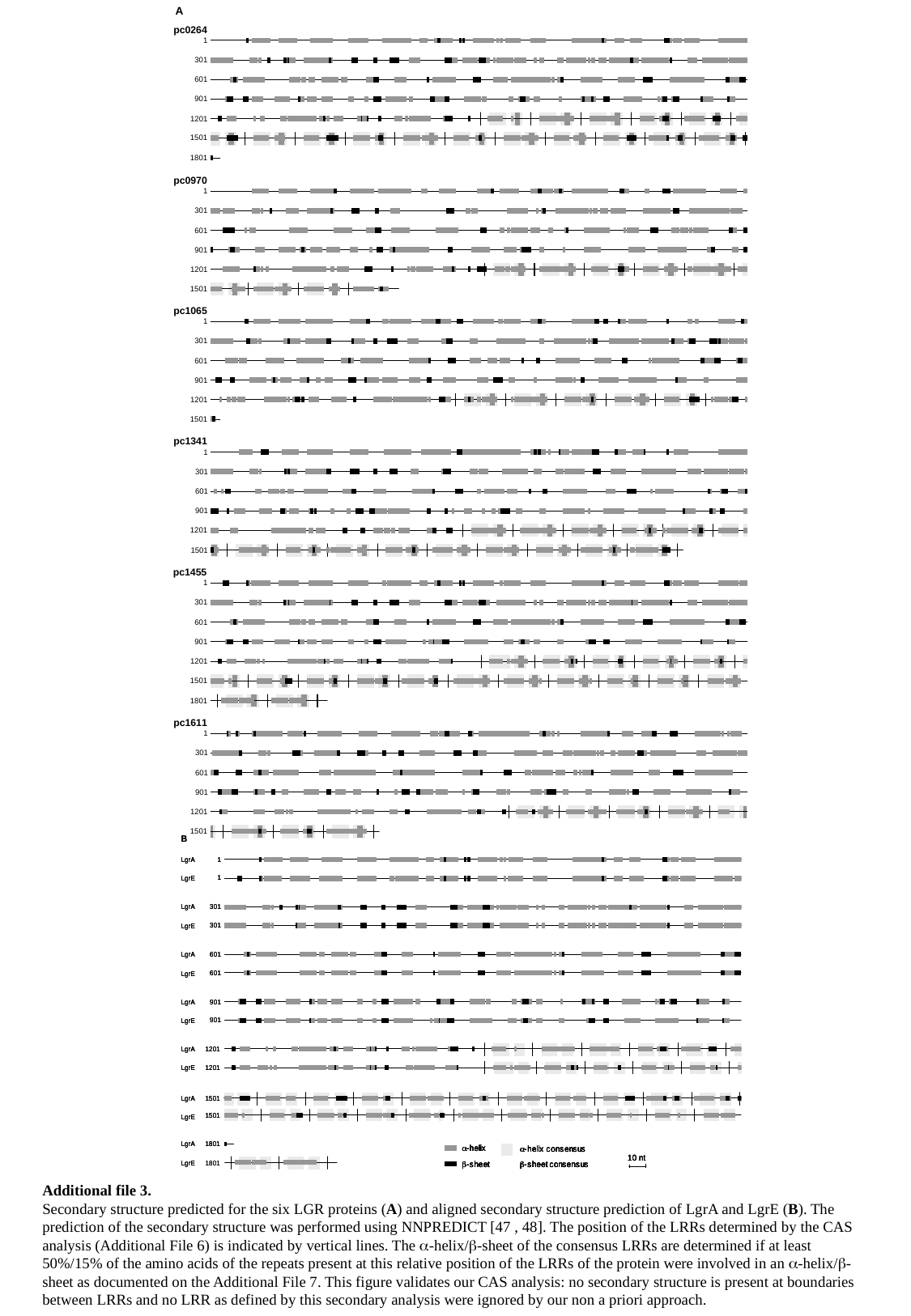

Additional file 3.
Secondary structure predicted for the six LGR proteins (A) and aligned secondary structure prediction of LgrA and LgrE (B). The prediction of the secondary structure was performed using NNPREDICT [47 , 48]. The position of the LRRs determined by the CAS analysis (Additional File 6) is indicated by vertical lines. The α-helix/β-sheet of the consensus LRRs are determined if at least 50%/15% of the amino acids of the repeats present at this relative position of the LRRs of the protein were involved in an α-helix/β-sheet as documented on the Additional File 7. This figure validates our CAS analysis: no secondary structure is present at boundaries between LRRs and no LRR as defined by this secondary analysis were ignored by our non a priori approach.
