# Supplementary material for: Analyses of six homologous proteins of Protochlamydia amoebophila UWE25 encoded by large GC-rich genes (lgr): a model of evolution and concatenation of leucine-rich repeats
Source: BMC Evol Biol. 2007 Nov 16;7:231. doi: 10.1186/1471-2148-7-231 (PMC2216083; doi:10.1186/1471-2148-7-231)
Supplement: Additional File 4 — Secondary structure of LGR proteins. The data shown in this table are the percentage of the amino acids predicted to be involved in α-helixes and β-sheets. [file 1471-2148-7-231-S4.doc]

# Additional file 4. Secondary structure of LGR proteins. The data shown are the percentage of the amino acids predicted to be involved in α-helixes and β-sheets. The analysis was done on the LRR and non-LRR domains, and on the complete protein sequence (LGR).

|  | α-helix | | | β-sheet | | |
| --- | --- | --- | --- | --- | --- | --- |
|  | LRR | non-LRR | LGR | LRR | non-LRR | LGR |
| LgrA | 45.2% | 45.2% | 45.2% | 8.1% | 6.9% | 7.2% |
| LgrB | 61.5% | 43.2% | 46.0% | 2.0% | 5.8% | 5.2% |
| LgrC | 50.6% | 44.9% | 45.5% | 6.5% | 7.0% | 7.0% |
| LgrD | 57.4% | 42.0% | 45.7% | 2.8% | 7.7% | 6.5% |
| LgrE | 46.8% | 46.6% | 46.7% | 3.1% | 6.7% | 5.7% |
| LgrF | 55.5% | 45.5% | 46.9% | 2.2% | 6.9% | 6.2% |
