# Supplementary material for: Analyses of six homologous proteins of Protochlamydia amoebophila UWE25 encoded by large GC-rich genes (lgr): a model of evolution and concatenation of leucine-rich repeats
Source: BMC Evol Biol. 2007 Nov 16;7:231. doi: 10.1186/1471-2148-7-231 (PMC2216083; doi:10.1186/1471-2148-7-231)
Supplement: Additional File 5 — Leucine content of the six LGR proteins. These analyses reveals no particular leucine enrichment of the LRR domain. [file 1471-2148-7-231-S5.ppt]

## Slide 1
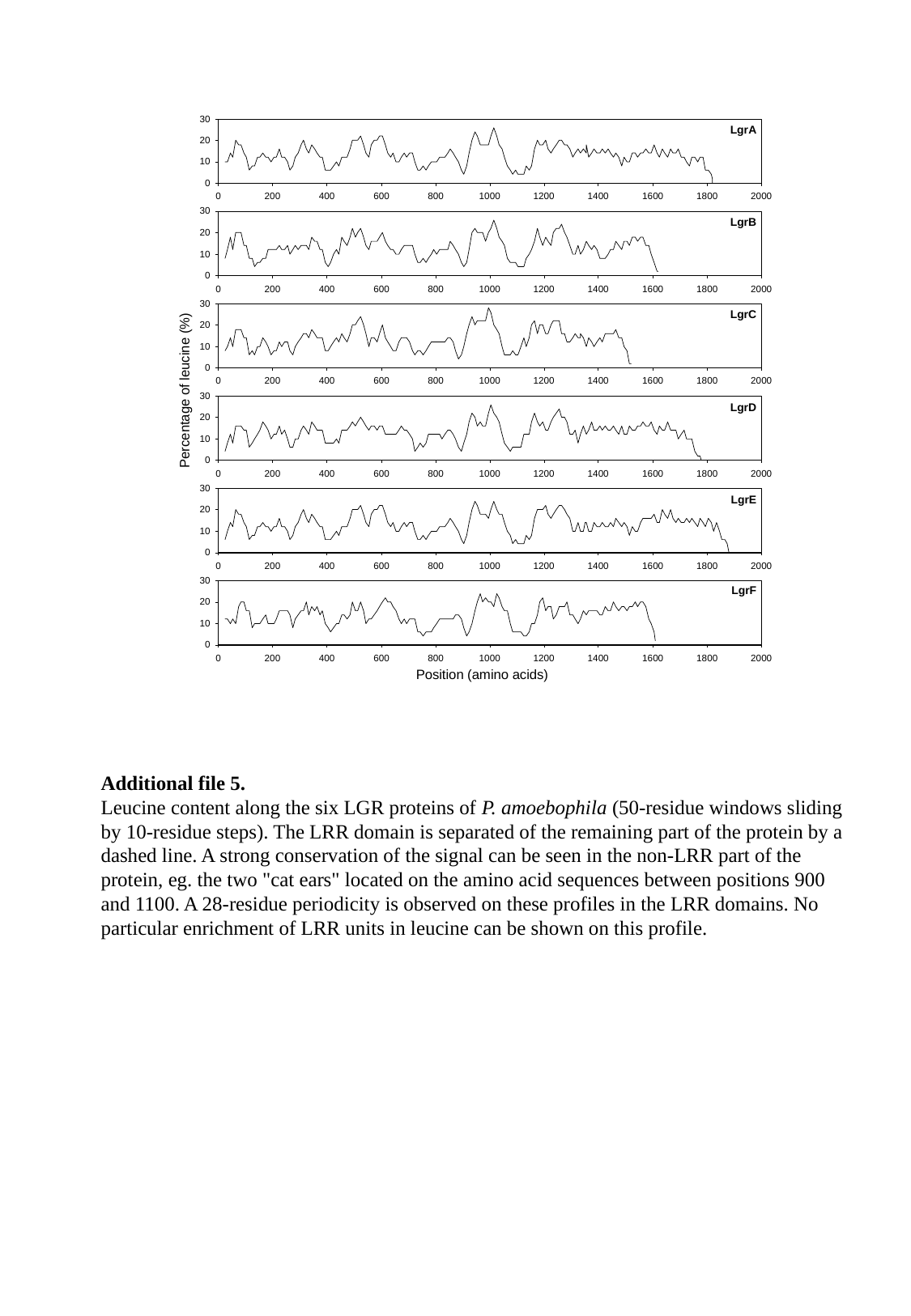

Additional file 5.
Leucine content along the six LGR proteins of P. amoebophila (50-residue windows sliding by 10-residue steps). The LRR domain is separated of the remaining part of the protein by a dashed line. A strong conservation of the signal can be seen in the non-LRR part of the protein, eg. the two "cat ears" located on the amino acid sequences between positions 900 and 1100. A 28-residue periodicity is observed on these profiles in the LRR domains. No particular enrichment of LRR units in leucine can be shown on this profile.
