# Supplementary material for: Analyses of six homologous proteins of Protochlamydia amoebophila UWE25 encoded by large GC-rich genes (lgr): a model of evolution and concatenation of leucine-rich repeats
Source: BMC Evol Biol. 2007 Nov 16;7:231. doi: 10.1186/1471-2148-7-231 (PMC2216083; doi:10.1186/1471-2148-7-231)
Supplement: Additional File 6 — Cumulative alignment score of the six LGR proteins. Representation of the cumulative alignment score of LgrA to LgrF. [file 1471-2148-7-231-S6.ppt]

## Slide 1
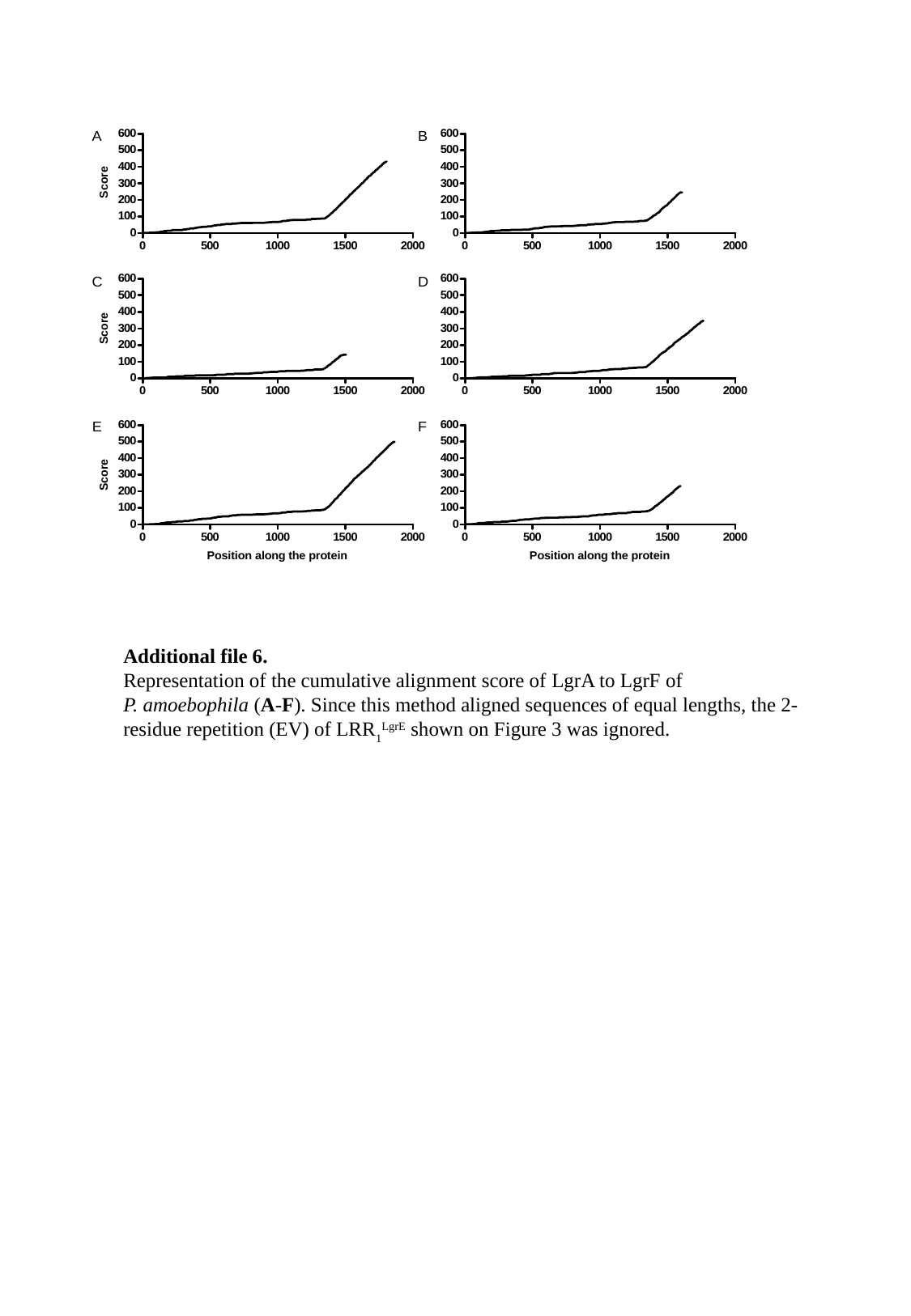

Additional file 6.
Representation of the cumulative alignment score of LgrA to LgrF of P. amoebophila (A-F). Since this method aligned sequences of equal lengths, the 2-residue repetition (EV) of LRR1LgrE shown on Figure 3 was ignored.
