# Supplementary material for: Analyses of six homologous proteins of Protochlamydia amoebophila UWE25 encoded by large GC-rich genes (lgr): a model of evolution and concatenation of leucine-rich repeats
Source: BMC Evol Biol. 2007 Nov 16;7:231. doi: 10.1186/1471-2148-7-231 (PMC2216083; doi:10.1186/1471-2148-7-231)
Supplement: Additional File 7 — Secondary structure of LRRs of the LGR proteins and related proteins. This figure shows the secondary structure of the LRRs of LgrA to LgrF of P. amoebophila, of the human NOD3 protein and of a LRR-protein of Legionella pneumophila. [file 1471-2148-7-231-S7.ppt]

## Slide 1
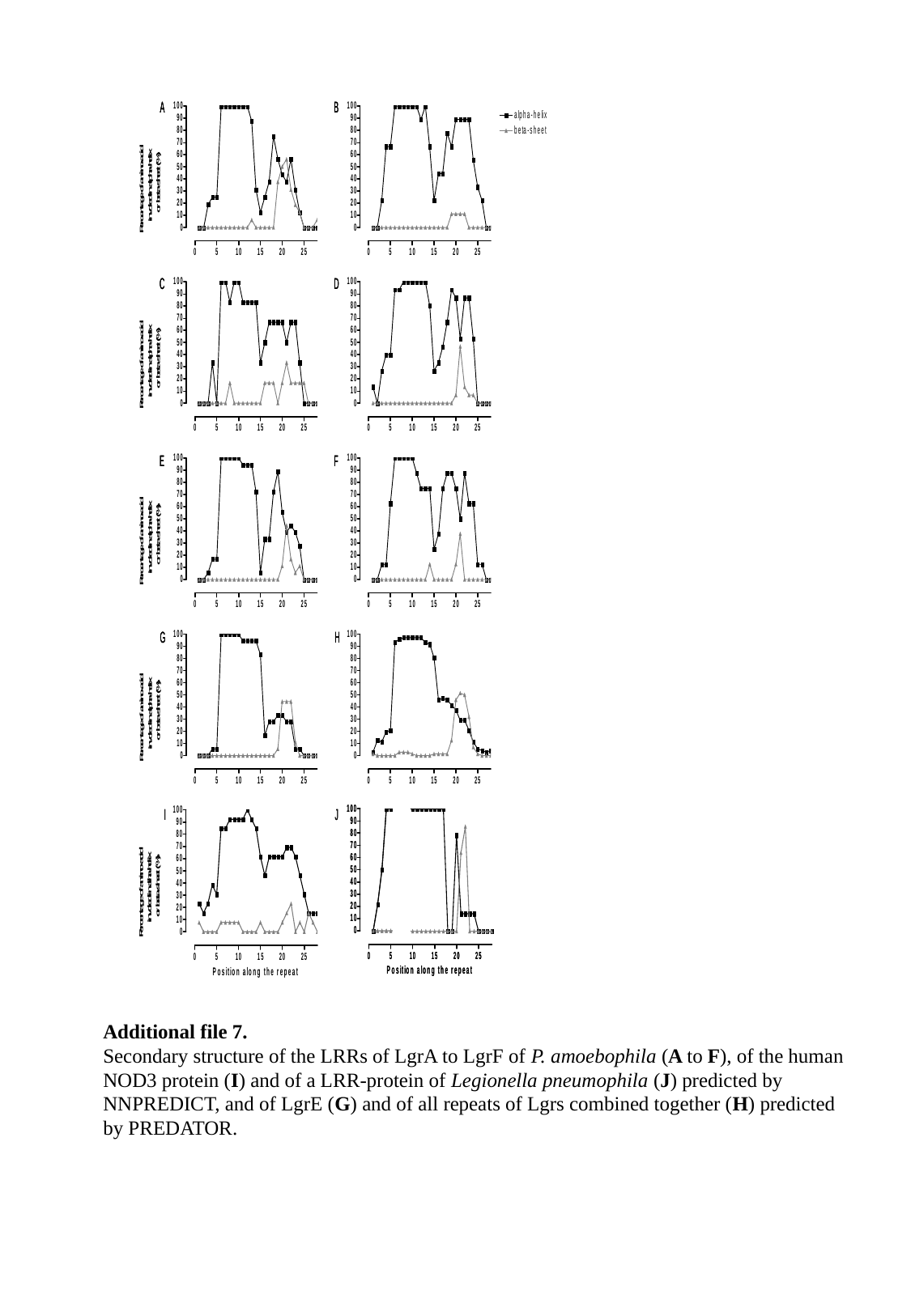

Additional file 7.
Secondary structure of the LRRs of LgrA to LgrF of P. amoebophila (A to F), of the human NOD3 protein (I) and of a LRR-protein of Legionella pneumophila (J) predicted by NNPREDICT, and of LgrE (G) and of all repeats of Lgrs combined together (H) predicted by PREDATOR.
