# Supplementary material for: Analyses of six homologous proteins of Protochlamydia amoebophila UWE25 encoded by large GC-rich genes (lgr): a model of evolution and concatenation of leucine-rich repeats
Source: BMC Evol Biol. 2007 Nov 16;7:231. doi: 10.1186/1471-2148-7-231 (PMC2216083; doi:10.1186/1471-2148-7-231)
Supplement: Additional File 8 — Phylogeny of the 72 LRRs related to LGRs. Phylogenetic analyses revealing that first and last repeats LRRs of LGRs proteins tend to cluster together. [file 1471-2148-7-231-S8.ppt]

## Slide 1
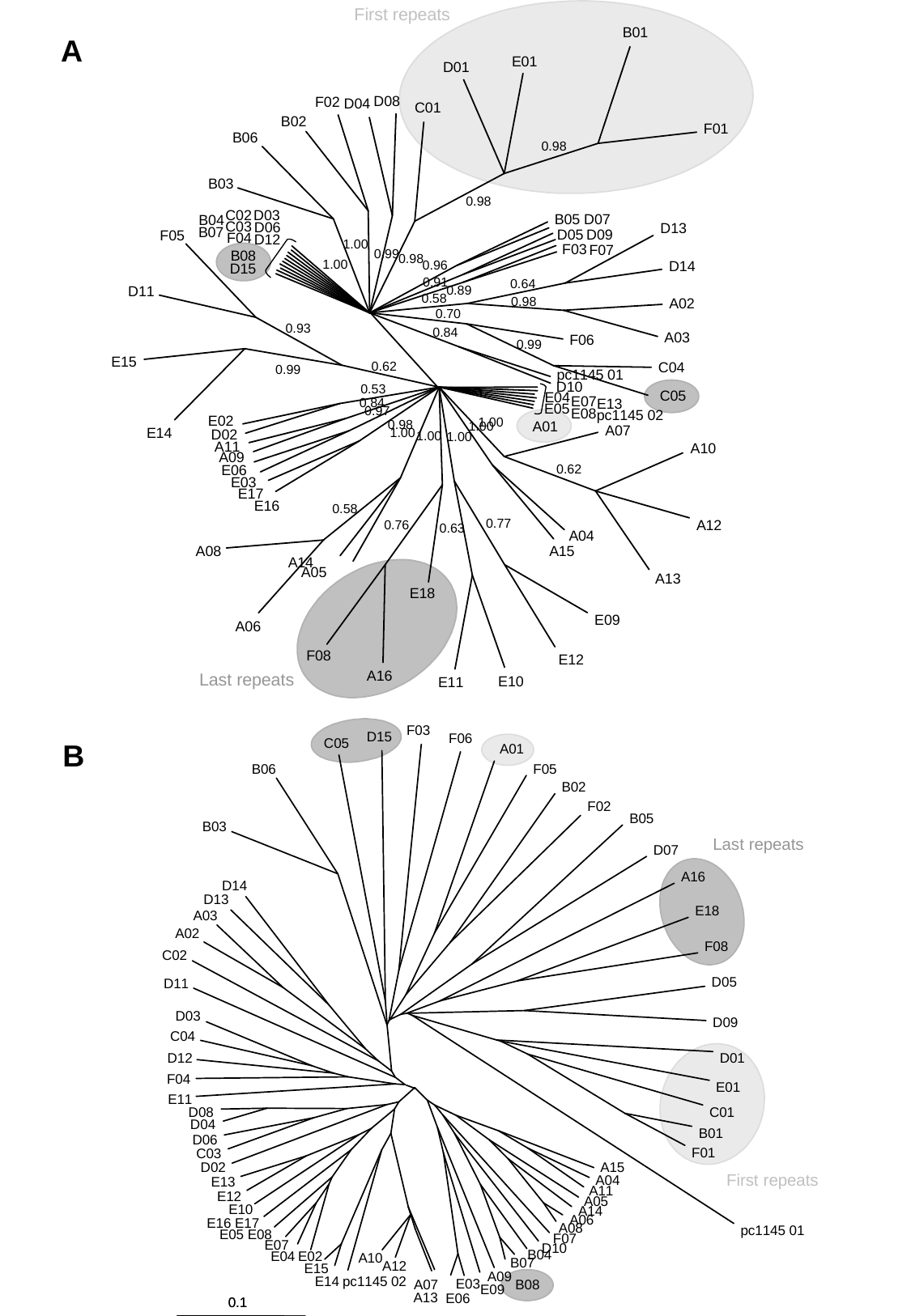

A
B

## Slide 2
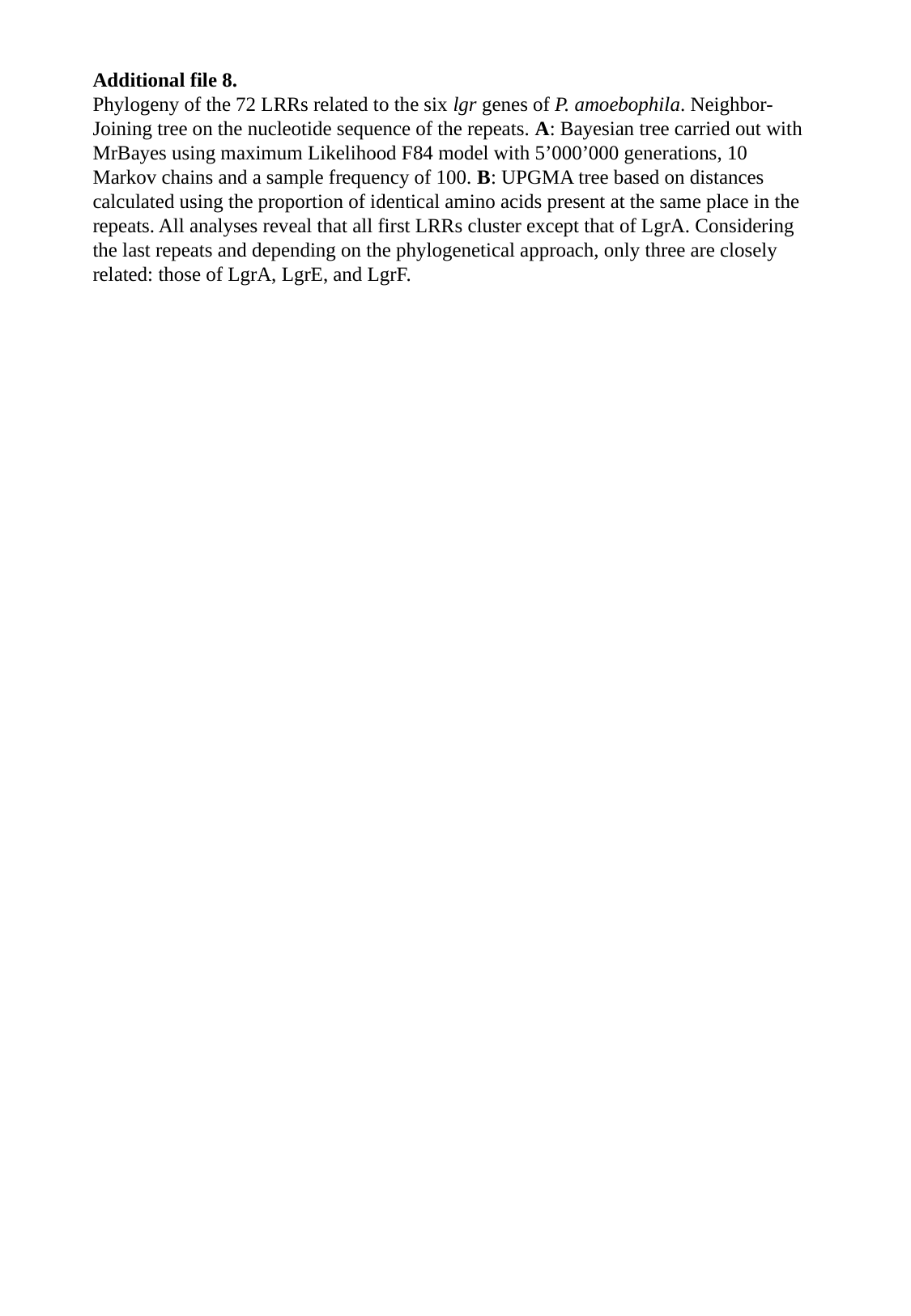

Additional file 8.
Phylogeny of the 72 LRRs related to the six lgr genes of P. amoebophila. Neighbor-Joining tree on the nucleotide sequence of the repeats. A: Bayesian tree carried out with MrBayes using maximum Likelihood F84 model with 5’000’000 generations, 10 Markov chains and a sample frequency of 100. B: UPGMA tree based on distances calculated using the proportion of identical amino acids present at the same place in the repeats. All analyses reveal that all first LRRs cluster except that of LgrA. Considering the last repeats and depending on the phylogenetical approach, only three are closely related: those of LgrA, LgrE, and LgrF.
