# Supplementary material for: Analyses of six homologous proteins of Protochlamydia amoebophila UWE25 encoded by large GC-rich genes (lgr): a model of evolution and concatenation of leucine-rich repeats
Source: BMC Evol Biol. 2007 Nov 16;7:231. doi: 10.1186/1471-2148-7-231 (PMC2216083; doi:10.1186/1471-2148-7-231)
Supplement: Additional File 9 — Identity scores between adjacent LRRs of the LGRs. These figures reveal that adjacent LRRs are closely related. [file 1471-2148-7-231-S9.ppt]

## Slide 1
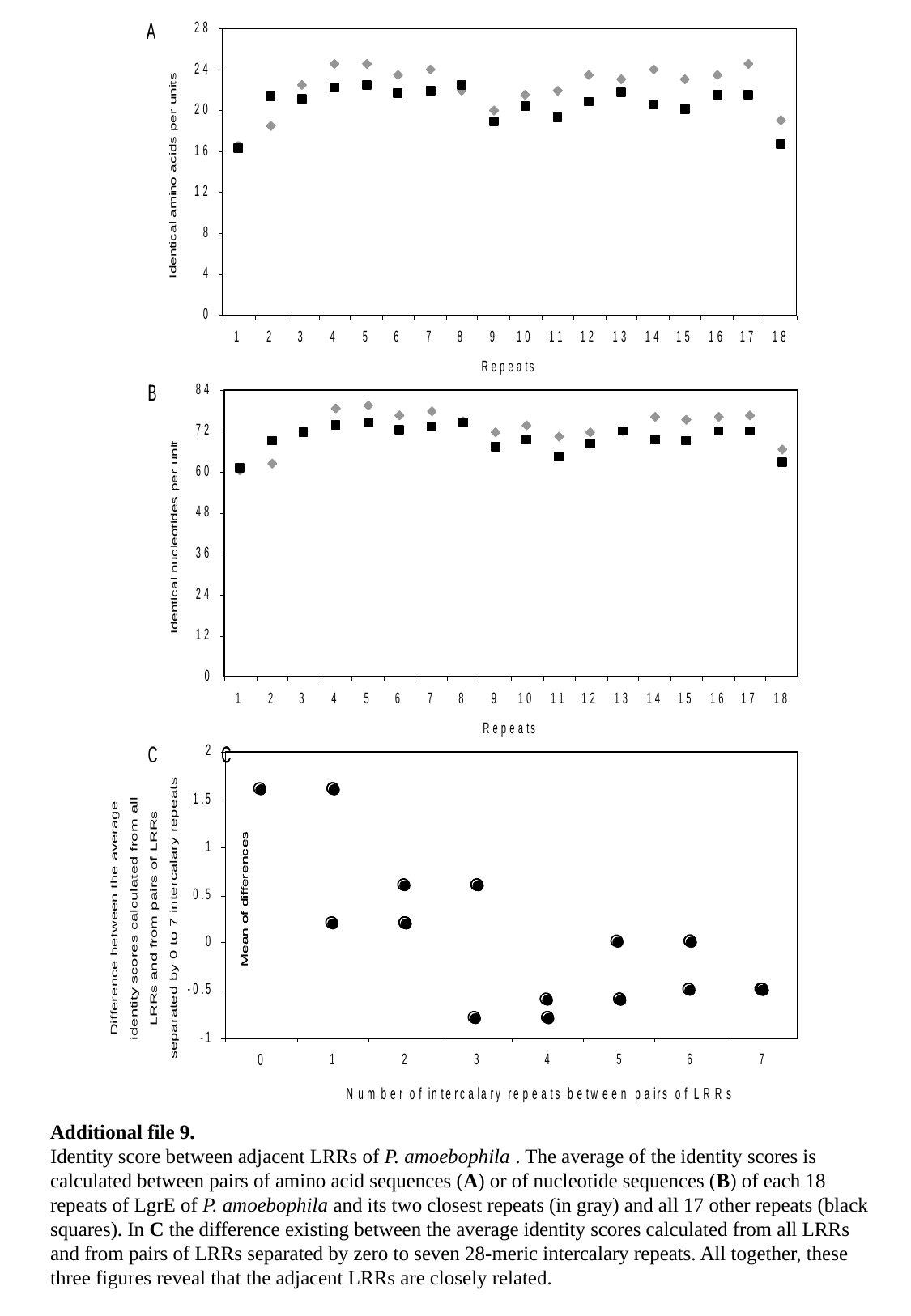

Additional file 9.
Identity score between adjacent LRRs of P. amoebophila . The average of the identity scores is calculated between pairs of amino acid sequences (A) or of nucleotide sequences (B) of each 18 repeats of LgrE of P. amoebophila and its two closest repeats (in gray) and all 17 other repeats (black squares). In C the difference existing between the average identity scores calculated from all LRRs and from pairs of LRRs separated by zero to seven 28-meric intercalary repeats. All together, these three figures reveal that the adjacent LRRs are closely related.
