# Supplementary material for: Analyses of six homologous proteins of Protochlamydia amoebophila UWE25 encoded by large GC-rich genes (lgr): a model of evolution and concatenation of leucine-rich repeats
Source: BMC Evol Biol. 2007 Nov 16;7:231. doi: 10.1186/1471-2148-7-231 (PMC2216083; doi:10.1186/1471-2148-7-231)
Supplement: Additional File 10 — Immediate neighborhood of lgrs. This figure shows the local genetic map of the six lgr genes of P. amoebophila. [file 1471-2148-7-231-S10.ppt]

## Slide 1
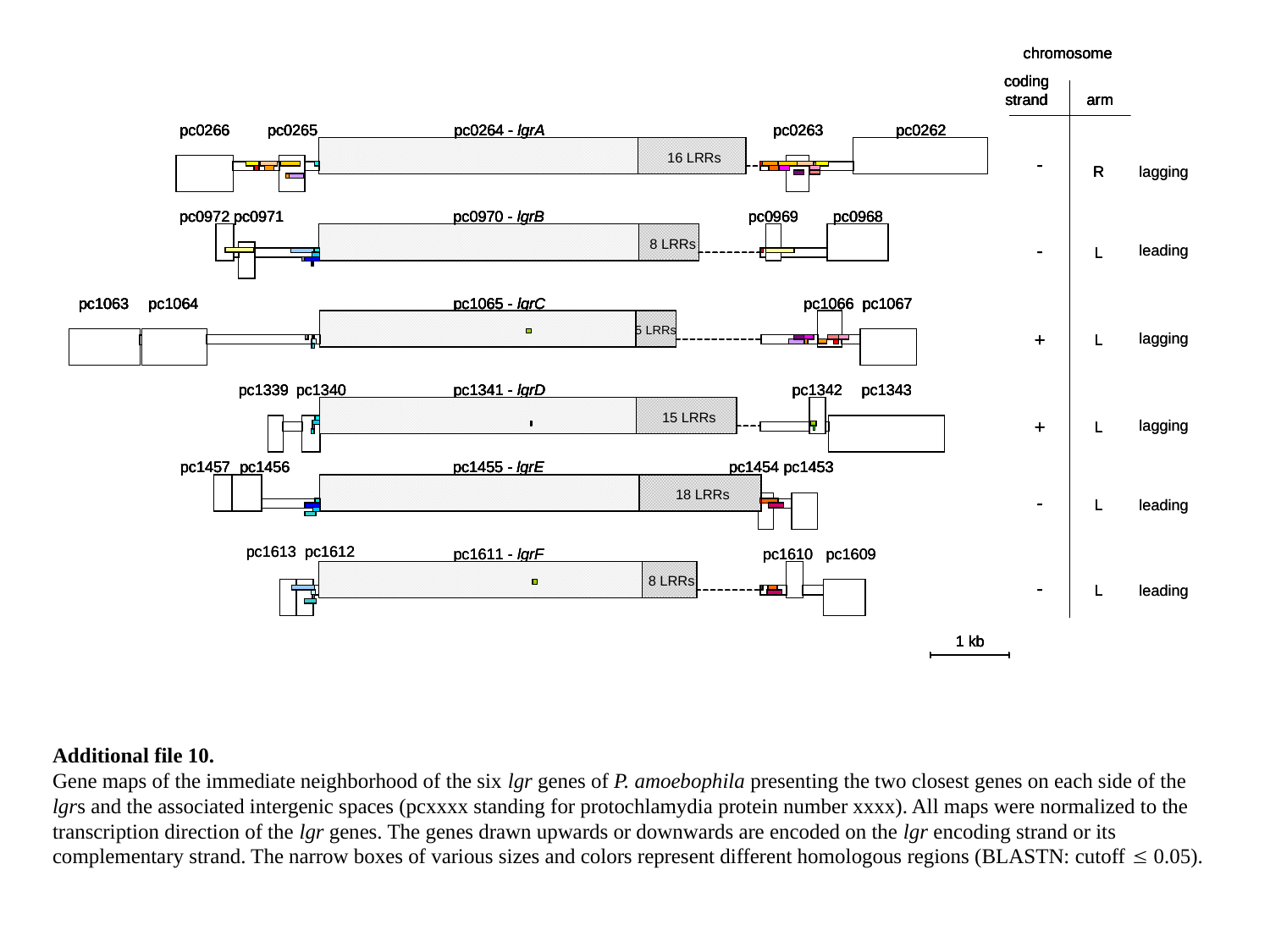

Additional file 10.
Gene maps of the immediate neighborhood of the six lgr genes of P. amoebophila presenting the two closest genes on each side of the lgrs and the associated intergenic spaces (pcxxxx standing for protochlamydia protein number xxxx). All maps were normalized to the transcription direction of the lgr genes. The genes drawn upwards or downwards are encoded on the lgr encoding strand or its complementary strand. The narrow boxes of various sizes and colors represent different homologous regions (BLASTN: cutoff ≤ 0.05).
