# Supplementary material for: Analyses of six homologous proteins of Protochlamydia amoebophila UWE25 encoded by large GC-rich genes (lgr): a model of evolution and concatenation of leucine-rich repeats
Source: BMC Evol Biol. 2007 Nov 16;7:231. doi: 10.1186/1471-2148-7-231 (PMC2216083; doi:10.1186/1471-2148-7-231)
Supplement: Additional File 11 — Putative promoter motifs of the six lgrs. A list of putative promoter motifs present upstream of the six lgr genes of P. amoebophila is provided in this table. [file 1471-2148-7-231-S11.doc]

**Additional file 11.** Putative promoter motifs of the 6 *lgr*s of *P. amoebophila.* While the underlined more conserved positions of canonical -35 and –10 motifs (TTG and TANNNT) are indicated in red and and the underlined ATG start codon is displayed in green.

| gene | distance* | G+C  content** | putative promoter motifs |
| --- | --- | --- | --- |
| *lgrA* | 38 | 16.0% | ATTTTAAATTTTTAAT**TTG**TTTTAGCTGTTTTTTTTGA**TA**GTA**T**CTTTACAACGTGAATTTTTGTT---AAATCAAAGGATAGCCAAGTCATG |
| *lgrB* | 38 | 22.0% | TTTTCAAATTTTAAA**TTG**ATTGGAGTGTTTCTTTTTGA**TA**GTA**T**CTTTACGACGTGAATTTTTGTT---AAATCAAAGGATAGCCAAGTCATG |
| *lgrC* | 35 | 24.0% | TTTTCAAATTTTAAA**TT**AATCAGGG-TATTTTTTT-GA**TA**TTG**T**TCTTTCAGCTCAAATTTCTTTATAAAAACCAAAGGATAGTCAATTCATG |
| *lgrD* | 38 | 20.0% | TTTTCAAATTTTAAA**TTG**ATTGAGGTATTTCTTTTTGA**TA**GTA**T**CTTTTCAATTTGAATTTTTGTT---AAATCAAAGGATAGCCAAGTCATG |
| *lgrE* | 36 | 18.0% | TTTTTCAAATTTAAA**TTG**ATTAGGGGTATTTTTTTTGC**TA**GTA**T**TTTTTTAACGTGAATTTT--TT---GTACCAAAGGATAGCCAAGTCATG |
| *lgrF* | 35 | 22.0% | TTTTCAAATTTTAAA**TTG**ATTAGGGATATTTTTTTTGC**TA**CTA**T**CCTTTCATTGCGAATTA------AAAAACAAAAAGATAGTCAAATCATG |
| *lgrB* | 156 | 32.0% | TAGCTTGAA-AGAATT**TTG**AATTGATCCCTTAGTGAGC**TA**GAT**T**TA-AAGCTTCAATACTTGAACCAAACTCTAGCAACCTAACTTTCTTTCC … |
| *lgrC* | 157 | 34.0% | TAGCTTGAT-AGAATT**TTG**GGTTGATCGATTAATAGGC**TA**GAT**T**CACAAGTTTTAACAATTAAACGAAATTCTAGCGATCTAAGTTTTTTCAC … |
| *lgrE* | 152 | 42.0% | TAGCTTGGTTAGGATT**TTG**GATTGACCTCTTAGTGCGC**TA**GAT**T**CA-AAGCTTCAACACTTGAACCAAACTTTAGCAACCTAAATTTCTCTCT … |
| *lgrF* | 153 | 34.0% | TAGCTTG---AGAATT**TTG**GATTGACCCCTTAGTGAGC**TA**AAT**T**TA-AAGCTGCAATACTTGAACCAAACTCTAGCAACATAATTTTCTCTCT … |

* distance between the transcription start site and the ATG start codon

** G+C content calculated in the 50 bp upstream of the transcription start site
