# Supplementary material for: Analyses of six homologous proteins of Protochlamydia amoebophila UWE25 encoded by large GC-rich genes (lgr): a model of evolution and concatenation of leucine-rich repeats
Source: BMC Evol Biol. 2007 Nov 16;7:231. doi: 10.1186/1471-2148-7-231 (PMC2216083; doi:10.1186/1471-2148-7-231)
Supplement: Additional File 12 — LRR consensus of the LGR proteins and of related proteins. Table listing proteins presenting an identity (BLASTP) of less than 10-50 with at least one of the LGR proteins and showing for each protein its LRR amino acid consensus. [file 1471-2148-7-231-S12.doc]

# Additional file 12. Characteristics of the LRR consensus of the proteins presenting an identity (BLASTP) of less than 10^-50^ with at least one of

# the LGR proteins. The more precisely the consensus is described, the more recent is the LRR domain.

| Protein name (accession number*) | Score of BLASTP with LgrE | Sequence identity | Length of protein (aa) | LRR region (AA position) | Consensus sequence of LRR** | | | Length of LRR *** | | Length of repeat | Number of repeats |
| --- | --- | --- | --- | --- | --- | --- | --- | --- | --- | --- | --- |
| Gala protein 1 *Ralstonia solanacearum* (CAD18065.1) | 6e-55 | 200/521 38% | 661 | 181-626 | IgxAG AQALAANTxLTSL DxxxNx | | | 446 | | 24 | 19 |
| Leucine-rich repeat protein *Ralstonia solanacearum* (BAD42392.1) | 3e-50 | 192/503 38% | 621 | 191-589 | IgxxG AxALAxxxxLxSL xLxxNx | | | 399 | | 24 | 17 |
| Hypothetical protein L*egionella pneumophila* str. Lens (CAH15819.1) | 2e-50 | 169/446 37% | 464 | 49-400 | IGDEG AKALAANQSLSTL NLSYNN | | | 352 | | 24 | 15 |
| LgrA *Protochlamydia amoebophila* UWE25 (YP_007263.1) | 0.0 | 1590/1805 88% | 1805 | 1352-1797 | ISDKGMEAFAQALASNTxLxxL SLNGNQ | | | 446 | | 28 | 16 |
| LgrB *Protochlamydia amoebophila* UWE25 (YP_007969.1) | 0.0 | 1153/1600 72% | 1605 | 1354-1592 | ISDEGAEAIAQALASNTTLxxL SLNxNQ | | | 239 | | 28 | 8 |
| LgrC *Protochlamydia amoebophila* UWE25 (YP_008064.1) | 0.0 | 1104/1505 73% | 1505 | 1338-1487 | ISDxGAEAxAQALxxNTALxxx xLxNNQ | | | 150 | | 28 | 5 |
| LgrD *Protochlamydia amoebophila* UWE25 (YP_008340.1) | 0.0 | 1257/1776 70% | 1764 | 1342-1752 | ISDKGAEAIAQALASNTxLxxL xLxNNQ | | | 411 | | 28 | 15 |
| LgrE *Protochlamydia amoebophila* UWE25 (YP_008454.1) | 0.0 | 1866/1866 100% | 1866 | 1352-1856 | ISDKGMEAFAQALASNTTLKSL xLxGNQ | | | 505 | | 28 | 18 |
| LgrF *Protochlamydia amoebophila* UWE25 (YP_008610.1) | 0.0 | 1194/1597 74% | 1594 | 1368-1592 | ISDKGxEALARALASNTALxxL xLNxNQ | | | 225 | | 28 | 8 |
| NOD3 *Canis familiaris* (XP_547153.2) | 1e-75 | 173/418 41% | 1151 | 759-1143 | IGxxGAxALAxALxxNxxLxxL xLQxNx | | | 385 | | 28 | 14 |
| NOD3 *Bos taurus* (XP_584462.2) | 5e-78 | 178/418 41% | 1194 | 802-1186 | IxxxGAxALAxALxxNxxLxxL xLQxNx | | | 385 | | 28 | 14 |
| NOD3 *Homo sapiens* (NP_849172.1) | 5e-72 | 178/418 41% | 1112 | 720-1104 | IxxxGAxAxAxALxxNxxLxxL xLQxNS | | | 385 | | 28 | 14 |
| FLJ00180 protein *Homo sapiens* (BAB84935.1) | 7e-69 | 178/418 41% | 499 | 107-491 | IxxxGAxALAxALxxNxxLxxL xLxxNx | | | 385 | | 28 | 14 |
| Caterpillar 16.2 Homo sapiens (AAT48367.1) | 7e-72 | 178/418 41% | 1065 | 673-1057 | IxxxGAxALAxALxxNxxLxxL xLQxNx | | | 385 | | 28 | 14 |
| NOD3 *Rattus norvegicus* (XP_220212.3) | 8e-71 | 173/427 40% | 1153 | 789-1145 | IGxxGAxxxAxALxxNxxLxxL xLQxNx | | | 357 | | 28 | 13 |
| mFLJ00348 protein *Mus musculus* (BAD90390.1) | 3e-67 | 169/418 40% | 1089 | 697-1081 | IGxxGAxALAxALxxNxxLxxL xLQxNx | | | 385 | | 28 | 14 |
| Unnamed protein product *Mus musculus* (BAC37747.1) | 1e-51 | 134/307 43% | 312 | 5-311 | IGxxGAxALAxALxxNxTLxxL xLQxNx | | | 307 | | 28 | 11 |
| Unnamed protein product *Mus musculus* (BAC39136.1) | 8e-62 | 160/386 41% | 397 | 5-389 | IGxxGAxALAxALxxNxTLxxL xLQxNx | | | 385 | | 28 | 14 |
| Caterpillar 16.2 isoform 1 *Mus musculus* (XP_484522.3) | 6e-54 | 137/336 40% | 343 | 36-342 | IGDxGAxALAxALxxNxTLxxL xLQxNx | | | 307 | | 28 | 11 |
| Caterpillar 16.2 isoform 3 *Mus musculus* (XP_889649.1) | 8e-67 | 169/418 40% | 1102 | 710-1094 | IGxxGAxALAxALxxNxxLxxL xLQxNx | | | 385 | | 28 | 14 |
| DlrA *Dictyostelium discoideum* (AAK76360.1) | 6e-49 | 124/404 30% | 817 | 376-761 | IGxxGxxxxxEALxxNxxIxxL xLSxNx | | | 386 | | 28 | 14 |
| Hypothetical protein DDB0220015 *Dictyostelium discoideum* (XP_635706.1) | 4e-49 | 124/404 30% | 818 | 376-761 | IGxxGxxxLxEALxxNxxIxxL xLSxNx | | | 386 | | 28 | 14 |
| Hypothetical protein DDB0205814 *Dictyostelium discoideum* (XP_641671.1) | 1e-53 | 145/492 29% | 617 | 110-463 | XxxxGxxxLAxxLxxNxxIxxL xLxxNx | | | 354 | | 28 | 13 |
| EMB2004 *Arabidopsis thaliana* (NP_563871.1) | 8e-59 | 152/556 34% | 605 | 186-563 | IxxxGAxxxAxxLKxNxxxxxL xLxxNx | | | 378 | | 28 | 14 |
| DlrA-like *Oryza sativa* (BAD37894.1) | 1e-56 | 146/423 34% | 628 | 240-598 | XxxxGxLxLxxxxxxNxxxxxL xLxxNx | | | 359 | | 28 | 13 |
| Hypothetical protein TTHERM_01557020 *Tetrahymena thermophila* SB210 (EAR81710.1) | 1e-51 | 177/520 34% | 623 | 7-615 | IGDKGAQNIGLGLSNCTQLTNLKFxIxxNx | | | 609 | | 30 | 20 |
| Hypothetical protein TTHERM_01054110 *Tetrahymena thermophila* SB210 (EAR82926.1) | 9e-50 | 170/533 31% | 1012 | 89-954 | IGDxGASxIGxALxNCKxLTNLxFxIxxNQ | | | 866 | | 30 | 29 |
| Hypothetical protein TTHERM_00630640 *Tetrahymena thermophila* SB210 (EAS02544.1) | 9e-50 | 185/608 30% | 690 | 6-642 | IGDDGASxIGxxLxNCKxLTNLxFxIxQNQ | | | 637 | | 30 | 21 |
| * accession number of protein as available on NCBI website [32] | | | | | |  |  | |  | |  |
| ** consensus sequence were aligned on *P. amoebophila* leucine-rich repeats | | | |  | |  |  | |  | |  |
| *** estimated with CAS (last repeat may be incomplete: the repeat was counted only when they measure at least the half length of the consensus) | | | | | |  |  | |  | |  |
