# Supplementary material for: Analyses of six homologous proteins of Protochlamydia amoebophila UWE25 encoded by large GC-rich genes (lgr): a model of evolution and concatenation of leucine-rich repeats
Source: BMC Evol Biol. 2007 Nov 16;7:231. doi: 10.1186/1471-2148-7-231 (PMC2216083; doi:10.1186/1471-2148-7-231)
Supplement: Additional File 13 — Phylogenetic analyses of the LRR domain of LGRs and related proteins. Phylogenetic analyses showing the relatedness of LRRs of LGRs with LRR proteins of proteobacteria and NODs of mammals. [file 1471-2148-7-231-S13.ppt]

## Slide 1
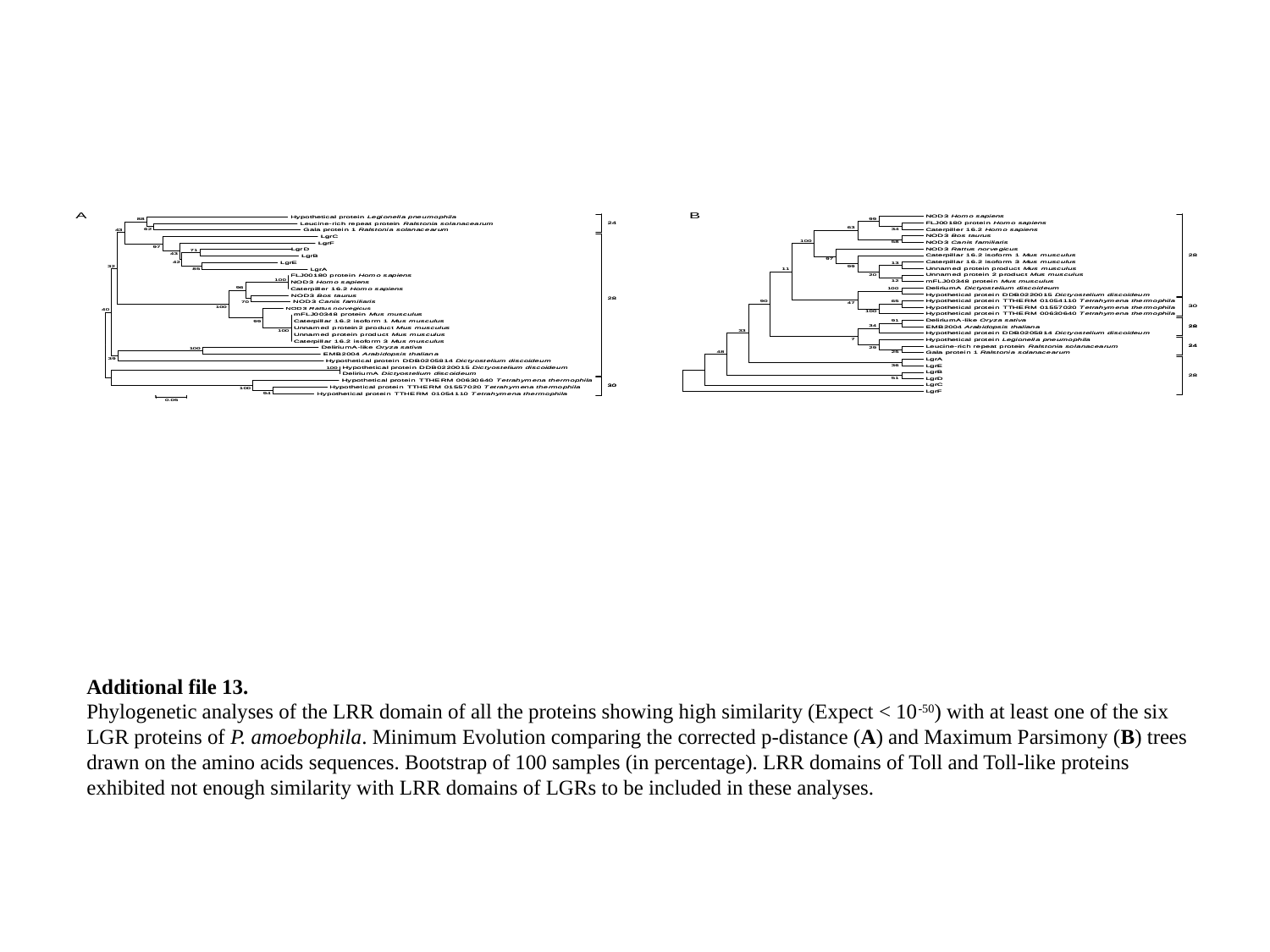

Additional file 13.
Phylogenetic analyses of the LRR domain of all the proteins showing high similarity (Expect < 10-50) with at least one of the six LGR proteins of P. amoebophila. Minimum Evolution comparing the corrected p-distance (A) and Maximum Parsimony (B) trees drawn on the amino acids sequences. Bootstrap of 100 samples (in percentage). LRR domains of Toll and Toll-like proteins exhibited not enough similarity with LRR domains of LGRs to be included in these analyses.
