# Supplementary material for: Analyses of six homologous proteins of Protochlamydia amoebophila UWE25 encoded by large GC-rich genes (lgr): a model of evolution and concatenation of leucine-rich repeats
Source: BMC Evol Biol. 2007 Nov 16;7:231. doi: 10.1186/1471-2148-7-231 (PMC2216083; doi:10.1186/1471-2148-7-231)
Supplement: Additional File 14 — Pfam analyses on all Lgrs. Putative functions of the conserved Pfam domains of LGRs are shown in this table. [file 1471-2148-7-231-S14.doc]

**Additional file 14**. Pfam analyses performed on all Lgrs. The domain and the putative function of the conserved Pfam domain are shown in the first and second columns. The E-value is reported for each LGR presenting a Pfam domain characterized by an E-value above the cutoff.

| **Domain** | **Putative function** | **LgrA** | **LgrB** | **LgrC** | **LgrD** | **LgrE** | **LgrF** |
| --- | --- | --- | --- | --- | --- | --- | --- |
| **LRR_1*** | **Bacterial recognition** | **45** | **60** | **150** | **48** | **31** | **60** |
| **PetN** | **Hydrophobic protein** | **0.18** | **0.18** | **0.18** | **0.18** | **0.18** | **0.18** |
| **Lipoprotein_3** | **Membrane anchor** | **0.1** | **0.54** |  |  |  |  |
| **DUF 2027** | **DNA mismatch repair** | **0.05** | **-** | **0.34** | **0.23** | **0.015** | **-** |
| **Psf1** | **Initiation of DNA replication** | **0.97** | **-** | **-** | **-** | **-** | **-** |
| **DUF 270** | **Unknown function** | **0.38** | **-** | **-** | **-** | **0.38** | **-** |
| **Rcd1** | **Genes regulation** | **-** | **0.27** | **-** | **-** | **-** | **-** |
| **HIRA_B** | **Histone regulatory protein** | **-** | **0.34** | **-** | **0.13** | **-** | **-** |
| **Phage_mat-A** | **Phage maturation protein and phage RNA replication** | **-** | **0.42** | **-** | **-** | **-** | **-** |
| **WIF** | **Wnp binding** | **-** | **0.94** | **-** | **-** | **-** | **-** |
| **TBPIP** | **Tat binding protein** | **-** | **0.18** | **-** | **-** | **-** | **-** |
| **Nucleo_LEF-12** | Late expression factors | **-** | **0.028** | **-** | **-** | **-** | **-** |
| **DUF 334** | **Unknown function** | **-** | **0.12** | **-** | **-** | **-** | **-** |
| **DUF 815** | **Unknown function** | **-** | **0.99** | **-** | **-** | **-** | **-** |
| **DUF 1356** | **Unknown function** | **-** | **0.53** | **-** | **-** | **-** | **-** |
| **RhoGEF** | **Guanine exchange factor** | **-** | **-** | **0.25** | **-** | **-** | **-** |
| **DUF 294** | **Putative nucleotidyltransferase** | **-** | **-** | **0.14** | **-** | **-** | **-** |
| **DUF 1631** | **Unknown function** | **-** | **-** | **0.94** | **-** | **-** | **-** |
| **Glyco_transf_10** | **Glycosyltransferase** | **-** | **-** | **-** | **0.74** | **-** | **-** |
| **GYF** | **Proline-binding domain** | **-** | **-** | **-** | **0.38** | **-** | **-** |
| **LytR_cpsA_psr** | **Transcriptional attenuator domain** | **-** | **-** | **-** | **0.7** | **-** | **0.7** |
| **DUF777** | **Unknown function** | **-** | **-** | **-** | **0.83** | **-** | **-** |
| **Adenine_glyco** | **Base excision repair enzyme** | **-** | **-** | **-** | **-** | **0.91** | **-** |
| **Sigma_54_AID** | **Bacterial transcription initiation factor** | **-** | **-** | **-** | **-** | **0.25** | **-** |
| **Sororin** | **Mediator of sister chromatid cohesion** | **-** | **-** | **-** | **-** | **0.97** | **-** |
| **BESS** | **DNA binding domain** | **-** | **-** | **-** | **-** | **-** | **0.92** |
| **Ipa_EvcA** | **Invasion plasmid antigen** | **-** | **-** | **-** | **-** | **-** | **0.31** |
| **Bromo_TP** | **DNA binding domain** | **-** | **-** | **-** | **-** | **-** | **0.054** |
| **DUF 1735** | **Unknown function** | **-** | **-** | **-** | **-** | **-** | **0.88** |

* All 6 LGRs exhibited three to four matches of the carboxy terminal end; only the best E-value is given; - : no match of this LGR with the corresponding domain.
